# Mapping the connectivity of serotonin transporter immunoreactive axons to excitatory and inhibitory neurochemical synapses in the mouse limbic brain

**DOI:** 10.1007/s00429-016-1278-x

**Published:** 2016-08-02

**Authors:** Arnauld Belmer, Paul M. Klenowski, Omkar L. Patkar, Selena E. Bartlett

**Affiliations:** 1 Translational Research Institute, Queensland University of Technology, Brisbane, Qld 4059 Australia; 2 Institute of Health and Biomedical Innovation (IHBI), Queensland University of Technology, Brisbane, Australia

**Keywords:** Serotonin, Neuromodulation, Synaptophysin, Gephyrin, PSD-95, 3D reconstruction

## Abstract

Serotonin neurons arise from the brainstem raphe nuclei and send their projections throughout the brain to release 5-HT which acts as a modulator of several neuronal populations. Previous electron microscopy studies in rats have morphologically determined the distribution of 5-HT release sites (boutons) in certain brain regions and have shown that 5-HT containing boutons form synaptic contacts that are either symmetric or asymmetric. In addition, 5-HT boutons can form synaptic triads with the pre- and postsynaptic specializations of either symmetrical or asymmetrical synapses. However, due to the labor intensive processing of serial sections required by electron microscopy, little is known about the neurochemical properties or the quantitative distribution of 5-HT triads within whole brain or discrete subregions. Therefore, we used a semi-automated approach that combines immunohistochemistry and high-resolution confocal microscopy to label serotonin transporter (SERT) immunoreactive axons and reconstruct in 3D their distribution within limbic brain regions. We also used antibodies against key pre- (synaptophysin) and postsynaptic components of excitatory (PSD95) or inhibitory (gephyrin) synapses to (1) identify putative 5-HTergic boutons within SERT immunoreactive axons and, (2) quantify their close apposition to neurochemical excitatory or inhibitory synapses. We provide a 5-HTergic axon density map and have determined the ratio of synaptic triads consisting of a 5-HT bouton in close proximity to either neurochemical excitatory or inhibitory synapses within different limbic brain areas. The ability to model and map changes in 5-HTergic axonal density and the formation of triadic connectivity within whole brain regions using this rapid and quantitative approach offers new possibilities for studying neuroplastic changes in the 5-HTergic pathway.

**Electronic supplementary material:**

The online version of this article (doi:10.1007/s00429-016-1278-x) contains supplementary material, which is available to authorized users.

## Introduction

Serotonin (5-hydroxytryptamine, 5-HT) is a neuromodulator extensively implicated in the regulation of mood, emotion, sleep and appetite. Furthermore, alterations in 5-HTergic neuronal signaling contribute to various neuropsychiatric disorders such as anxiety, major depression and drug abuse. Serotonin neurons arising from the brainstem raphe nuclei send their projections throughout the brain to form direct synapses with the neuropil of the targeted neurons. In addition, 5-HT is diffusely released by “volume transmission” (extra-synaptic) in several brain regions and initiates neuromodulatory activity of excitatory and inhibitory synapses in contrast to “classical” neurotransmitters (Bunin and Wightman 1998; De-Miguel and Trueta 2005; Kiss 2008). In some cases, 5-HT neurons contact glutamatergic or GABAergic/glycinergic synapses to form synaptic triads that modulate the activity of excitatory or inhibitory synapses (For review, see Ciranna 2006).

The distribution of 5-HT release sites along the varicose axons (boutons) in the brain has primarily and extensively been determined from electron microscopy studies in rats (for review, see Descarries et al. 2010). Furthermore, immunocytochemistry and autoradiography studies have shown that two distinct morphological types of 5-HTergic varicose axons densely innervate all brain regions and make both symmetrical (excitatory) and asymmetrical (inhibitory) synapses with variable synaptic incidences (for review, see Descarries et al. 2010). These studies have also revealed a variable distribution of 5-HTergic contact types (i.e., symmetric, asymmetric or undefined) throughout the brain, suggesting that the excitatory/inhibitory balance of 5-HTergic innervation is finely tuned in discrete regions. While both excitatory and inhibitory synaptic triads have also been demonstrated in these studies, the labor intensive batch processing of serial sections and multistep immunolabeling required to identify neurochemical markers from electron microscopy sections makes their quantitative distribution throughout the brain poorly understood. Furthermore, species differences (rat, monkey, cat, and guinea pig) and the use of different labeling techniques and markers for 5-HTergic axons [antibodies directed against 5-HT, the serotonin transporter (SERT), the synthesis enzyme tryptophan hydroxylase 2 (TPH2), or radiolabeling with [3H] 5-HT] have made differences in 5-HT axonal distribution and 5-HTergic triadic connectivity difficult to interpret.

Recently, improved automated or semi-automated techniques have been developed for in vitro and in vivo quantification of synapses using fluorescence microscopy (Ippolito and Eroglu 2010; Schätzle et al. 2012; Dumitriu et al. 2012; Busse and Smith 2013; Fogarty et al. 2013; Danielson and Lee 2014; Sanders et al. 2015; Klenowski et al. 2015; Sigal et al. 2015). The combination of fluorescence microscopy, immunolabeling of key pre- and postsynaptic markers of excitatory/inhibitory synapses and automated software analysis, has afforded new high-throughput methodology allowing for rapid quantification of putative neurochemical synapses. Similar approaches have also been used to characterize the architecture of excitatory and inhibitory inputs onto TPH-positive neurons in the dorsal raphe (DR) and identify GABA-glutamate synaptic triads that may control excitatory transmission in the DR (Soiza-Reilly et al. 2013; Soiza-Reilly and Commons 2014).

Here, we have adapted a semi-automated method to quantify the 3D distribution of serotonergic axons, determine the number of putative serotonergic boutons, and quantify those in close apposition to neurochemical excitatory or inhibitory synapses in different regions and subregions of the mouse limbic brain including the medial prefrontal cortex (mPFC), the nucleus accumbens shell (NACs) and core (NACc), bed nucleus of the stria terminalis (BNST), the basolateral (BLA) and central (CeA) amygdala, the hippocampus (HIP) and the ventral tegmental area (VTA). We used an antibody against the serotonin transporter SERT, which has been shown to be a more robust marker of 5-HT axons than 5-HT itself (Nielsen et al. 2006), to reconstruct 5-HTergic axons using IMARIS software which allowed for volumetric quantification of SERT-immunoreactive (SERT^+^) axon fiber density in these brain regions. Concurrently, we identified putative serotonergic presynaptic release sites (boutons) within the SERT^+^ axons and quantified 5-HTergic boutons in close apposition to neurochemical excitatory and inhibitory synapses. We used well validated and commercially available antibodies to immunolabel key pre- and postsynaptic specializations of excitatory and inhibitory synapses. For SERT^+^ boutons and presynaptic endings of both excitatory (glutamate) and inhibitory (GABA/glycine) terminals, we used an antibody against synaptophysin (SYN), an abundant synaptic vesicle membrane protein involved in synaptic vesicle exocytosis (McMahon et al. 1996). For excitatory postsynaptic specializations, we have used an antibody against postsynaptic density 95 (PSD95), a scaffolding protein that interacts with glutamate receptors and dendritic spine cytoskeletons in the postsynaptic membrane of neurons (Sheng and Pak 2000; Sheng and Sala 2001). For inhibitory postsynaptic specializations, we have used an antibody against gephyrin (GEPH), a scaffolding protein that interacts with postsynaptic GABAergic and glycinergic receptors (for review, see Tyagarajan and Fritschy 2014). Confocal images were then acquired and used to perform semi-automated spot analysis (Fogarty et al. 2013; Klenowski et al. 2015) using Imaris 8.1.2 software. This involved isolating the SYN puncta within SERT^+^ axons (SYN^SERT+^ boutons) that were located within a distance of 0.6 μm to either the pre- or postsynaptic components of neurochemical excitatory (excitatory triads) or inhibitory synapses (inhibitory triads). This allowed us to quantify and map the distribution of serotonergic excitatory and inhibitory triads within the limbic brain structures.

This work provides a robust and reproducible quantitative method of rapidly screening the SERT^+^ axonal density and serotonergic triadic connectivity in the mouse brain. The use of this methodology in the future studies could help to uncover changes in 5-HT neuronal fiber density and 5-HT connectivity in animal models of neuropsychiatric disorders and drug addiction.

## Materials and methods

### Animals

Male C57BL/6J mice (*n* = 3, 5-week-old, ARC, WA, Australia), were group-housed in ventilated Plexiglas cages with ad libitum access to food and water in a climate-controlled, 12-h light/dark cycle room (lights on at 9:00 am). Mice were given 1 week to acclimatize to the housing conditions prior to the start of experiments. All procedures were approved by the Queensland University of Technology animal ethics committee and the University of Queensland animal ethics committee.

### Fixation and tissue processing

Animals were transcardially perfused with 4 % paraformaldehyde in 0.1-M phosphate-buffered saline solution (PBS), pH 7.4. Brains were dissected, removed and postfixed overnight. They were then cryoprotected in 20 % sucrose in PBS for 24 h at 4 °C followed by an overnight incubation in 30 % sucrose in PBS at 4 °C. Brains were embedded in optimal cutting temperature (OCT, Tissue-Tek, ProSciTech, Australia), snap frozen in a dry ice isopentane slurry and kept at −80 °C until processing. Brains were serially sectioned (30 μm) in the coronal plane using a cryostat (ThermoScientific HM525NX) and kept as free-floating sections in ice-cold PBS. Sections were rinsed 3 times in PBS to remove any trace of OCT.

### Antibodies and sera

Rabbit polyclonal anti-SERT antibody (PC177L) and mouse monoclonal anti-synaptophysin antibody (MAB5258) were purchased from Merck Millipore (Bayswater, Australia). Mouse monoclonal anti-PSD95 antibody (6G6-1C9) and rabbit polyclonal anti-gephyrin (Ab32206) antibody were purchased from Abcam (Melbourne, Australia). Goat anti-rabbit-Alexa488, goat anti-rabbit Alexa555, goat anti-mouse-Cy5, and goat anti-mouse-Alexa405 antibodies were purchased from Life Technologies (Mulgrave, Australia). Normal mouse (NMS), normal rabbit (NRS), normal goat (NGS) sera, goat F(ab) anti-mouse and anti-rabbit antibodies were purchased from Abcam (Melbourne, Australia) (Table 1).

**Table 1:** Antibodies used in this study

Antigen	Host	Immunogen	Supplier	Catalog #	Dilution	References
SERT	Rabbit	Synthetic peptide corresponding to amino acids 602–622 of rat 5-HT transporter	Millipore	PC177L	1/1000	Zhou et al. (1996)
Synaptophysin	Mouse	Vesicular fraction of bovine brain	Millipore	MAB5258	1/500	Tabuchi et al. (2007)
PSD95	Mouse	Purified recombinant rat PSD-95	Abcam	Ab2723	1/1000	Tomer et al. (2014)
Gephyrin	Rabbit	Synthetic peptide conjugated to KLH derived from within residues 700 to the C-terminus of Mouse Gephyrin	Abcam	Ab32206	1/1000	Nunez-Para et al. (2013)

### Immunohistochemistry

Specificity and optimal signal-to-noise ratios of each antibody were individually controlled by comparing the labeling produced by incubation of the secondary antibodies alone (Supp Fig. S1). Sequential immunohistochemical staining was then performed in two stages as follows: sections were first incubated at room temperature for 1 h in blocking solution [4 % NGS, 1 % bovine serum albumin (BSA), 0.3 % Triton and 0.05 % Tween20 in PBS]. Sections were then incubated for 24 h at 4 °C under orbital agitation in a mix of rabbit anti-SERT (1/1000) and mouse anti-PSD95 (1/1000) antibodies diluted in blocking solution. After 3 × 10 min washes in blocking solution, slices were incubated in a combination of goat anti-rabbit-Alexa488 (1/1000) and goat anti-mouse-Cy5 (1/1000) diluted in blocking solution for 4 h at room temperature. Slices were then washed in blocking solution (3 × 10 min) followed by PBS (3 × 10 min). To block the free binding sites from the first labeling round, slices were incubated in a mix of 5 % NMS and 10 % NRS diluted in blocking solution, for 1 h at room temperature under orbital agitation then rinsed 3 × 10 min in blocking solution. Slices were then incubated in goat anti-mouse (1/100) and goat anti-rabbit (1/100) monovalent F(ab) antibody fragments diluted in PBS and agitated for 1 h at room temperature, then washed 3 × 10 min in blocking solution. For the second stage of the immunolabeling process, slices were incubated in a combination of rabbit anti-gephyrin (1/1000) and mouse anti-synaptophysin (1/500) diluted in blocking solution for 24 h at 4 °C under agitation. After three washes in blocking solution, slices were incubated in a mixture of goat anti-rabbit-Alexa455 (1/1000) and goat anti-mouse-Alexa405 (1/750) diluted in blocking solution for 4 h at room temperature. Slices were then washed in blocking solution (3 × 10 min) followed by PBS (3 × 10 min) and mounted on slides with Prolong Gold antifade mouting media (Life Technologies, Scoresby, Australia).

### Control of specificity for second stage immunolabeling

To assess the specificity of the second round of primary antibodies, control slices were processed under the same conditions as above except that the second stage primary antibodies (anti-gephyrin and anti-synaptophysin) were omitted (Supp Fig. S2). To eliminate any bias in the labeling sequence, we interchanged the sequence of primary antibodies as well as the secondary antibody used to reveal each primary antibody.

### Imaging

Confocal images (1024 × 1024) were acquired on an Olympus FV1200 microscope fitted on a IX83 automated inverted platform with a 60× oil-immersed objective (NA 1.35) equipped with solid state lasers (405, 488, 559, and 635 nm) at an exposure of 2 µs/pixel, a numerical zoom of 2.5× and a *z*-step of 0.3 µm. Sequential scanning of the channels was used to avoid any overlapping in the emission/excitation wavelengths (488 + 635 and 405 + 541 nm). A total of 30 images at a *z*-depth of 20 µm (0.3-µm step size, 65 *z*-stacks) were acquired for each brain region. This combination yielded a pixel size of 82 µm with a resolution limit of 150 µm according to Abbe’s law (Wang and Smith 2012). To compensate for light scattering and the point spread function, images were deconvolved using Huygens Professional Software (SVI) with a maximum of 100 iterations, a quality threshold of 0.001 and a signal-to-noise ratio (SNR) of 10. Deconvolved images were saved as Imaris classic files to conserve the *x*/*y*/*z* voxel size. Deconvolution using Huygens has been shown to allow for the reconstruction of images with a resolution of 50 nm (lateral)/100 nm (axial) (Schrader et al. 1996).

### Image analysis using Imaris 8.1.2

Imaris analysis was performed as previously described (Schätzle et al. 2012; Fogarty et al. 2013; Klenowski et al. 2015). Briefly, SERT immunolabeled fibers were reconstructed in 3D using the surface rendering function in Imaris and the volumetric density was calculated. The ‘masking’ function was then used to remove intra-fiber labeling and conserve the synaptophysin (Syn^out^), PSD95 (PSD95^out^) and gephyrin (Geph^out^) punctuate fluorescence located outside the created SERT-surface, which was used to identify putative excitatory and inhibitory neurochemical synapses. In parallel, the removal of the synaptophysin fluorescence signal outside of the SERT-surface ensured that only intra-fiber synaptophysin labeling remained, allowing for quantification of putative 5-HT synaptic boutons (SYN^SERT+^). Then, the ‘spot detection’ function was individually applied for each created mask (Syn^in^: 5-HT bouton, Syn^out^/PSD95^out^: excitatory synapse and Syn^out^/Geph^out^: inhibitory synapse) for detection of puncta with a diameter of 0.4 µm and above. This size was selected based on the *z* step-size to ensure that puncta were present in a minimum of two confocal optical slices, as previously described (Fogarty et al. 2013; Klenowski et al. 2015). Localization of Syn^out^/PSD95^out^ or Syn^out^/Geph^out^ pairs within a distance of a 0.6 µm was performed using the ‘spot colocalization’ ImarisXT plugin to identify the appositions of pre- and postsynaptic markers as putative excitatory or inhibitory synapses, respectively. Then, SYN^SERT+^ boutons that were located within 0.6 µm of Syn^out^/PSD95^out^ (putative excitatory synapse) or Syn^out^/Geph^out^ (putative inhibitory synapse) spots pairs identified serotonergic excitatory and inhibitory triads, respectively. Data were plotted in Graphpad Prism 6.0 software (La Jolla, California, USA).

### Statistical analysis

Thirty images from three animals were averaged to generate a group mean (*n* = 3) and SEM. Statistical significance was assessed by one- or two-way ANOVA analysis of variances with Bonferroni post hoc multiple comparisons. All numerical data are expressed as mean ± SEM and significance was established at *p* < 0.05. To determine whether the proportion of putative excitatory/inhibitory synapses contributed to the number excitatory/inhibitory triads identified in each brain region, we performed Pearson correlation analysis on their respective ratios within the brain areas analyzed.

## Results

**Fig. 1:**
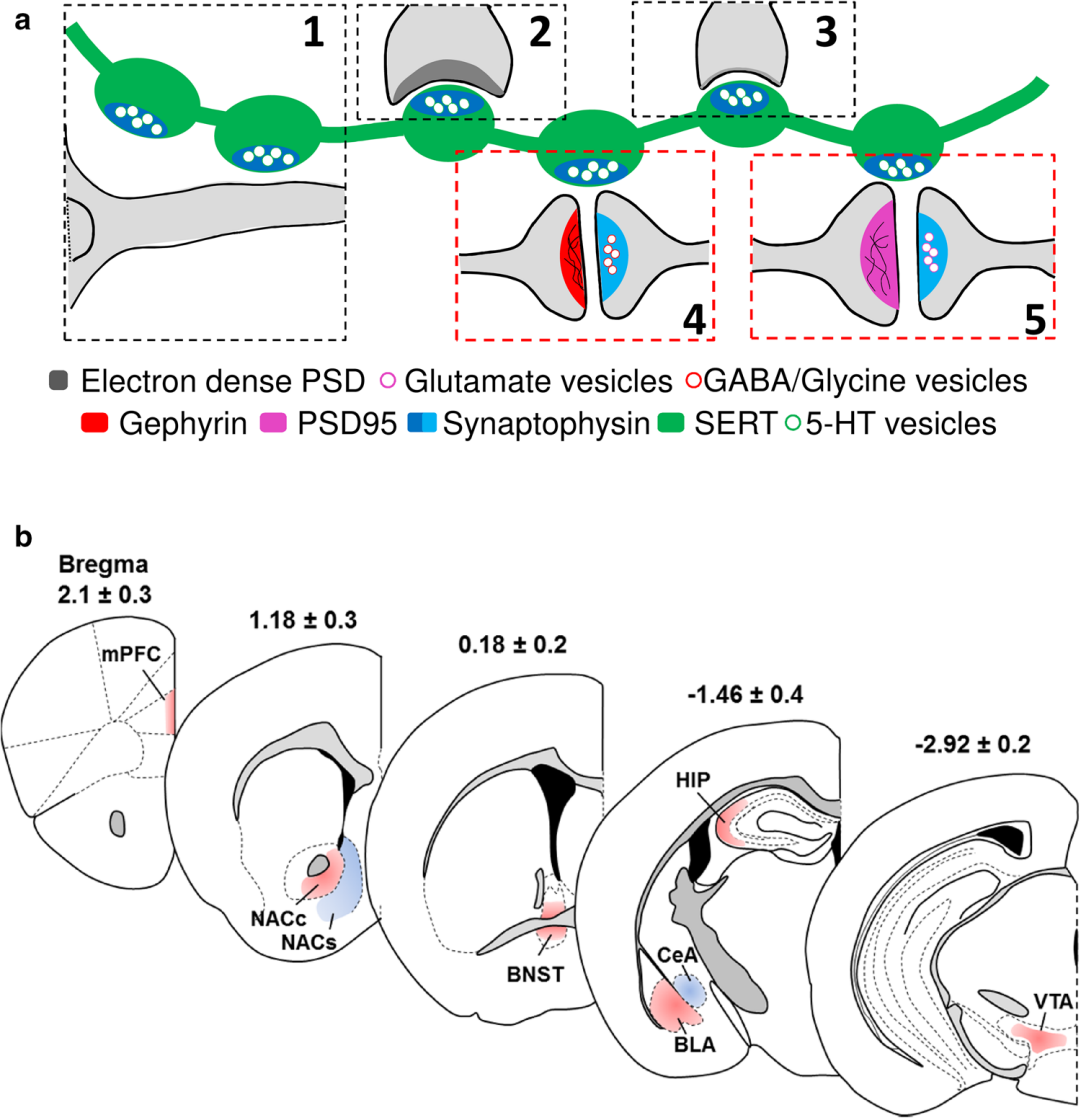
Overview of serotonergic connectivity in limbic brain regions. **a** Synaptic, extra-synaptic and triadic connectivity of 5-HT axons. 5-HT is released from varicosities of serotonergic axons both extra-synaptically (*1*) and synaptically into asymmetrical (*2*) and symmetrical synapses (*3*). In addition, serotonin varicosities appose excitatory/glutamate or inhibitory/GABA/glycine synapses to form triads consisting of a 5-HTergic bouton and the pre- and postsynaptic components of an excitatory or inhibitory synapse (*4*, *5*). In our study, putative 5-HT boutons were defined by synaptophysin puncta located inside the SERT immunoreactive axons (dark blue). Neurochemical excitatory or inhibitory synapses were defined as spot pairs consisting of the presynaptic marker synaptophysin puncta located outside of the SERT immunoreactive fibres (*light blue*) that were within 0.6 μm of the postsynaptic markers of excitatory (PSD95, purple) or inhibitory (gephyrin, *red*) postsynaptic markers synapses (*4*, *5*). Then, synaptic 5-HT triads were defined by putative 5-HT boutons in close apposition to either the pre- or postsynaptic marker of putative excitatory or inhibitory synapses (*4*, *5*). **b** Coronal diagrams of limbic brain regions selected for the study included layer I/III of the prelimbic area of the medial prefrontal cortex (mPFC), the ventral core and medial shell of the nucleus accumbens (NACc and NACs, respectively), the ventromedial and posterolateral part of the bed nucleus of the stria terminalis (BNST), the central and basolateral amygdala (CeA and BLA, respectively), the *strata oriens* layer of the CA3 region of the hippocampus (HIP), and the ventral tegmental area (VTA). The coordinates corresponding to each brain region (*top* of *each diagram*) are relative to bregma (mm)

**Fig. 2:**
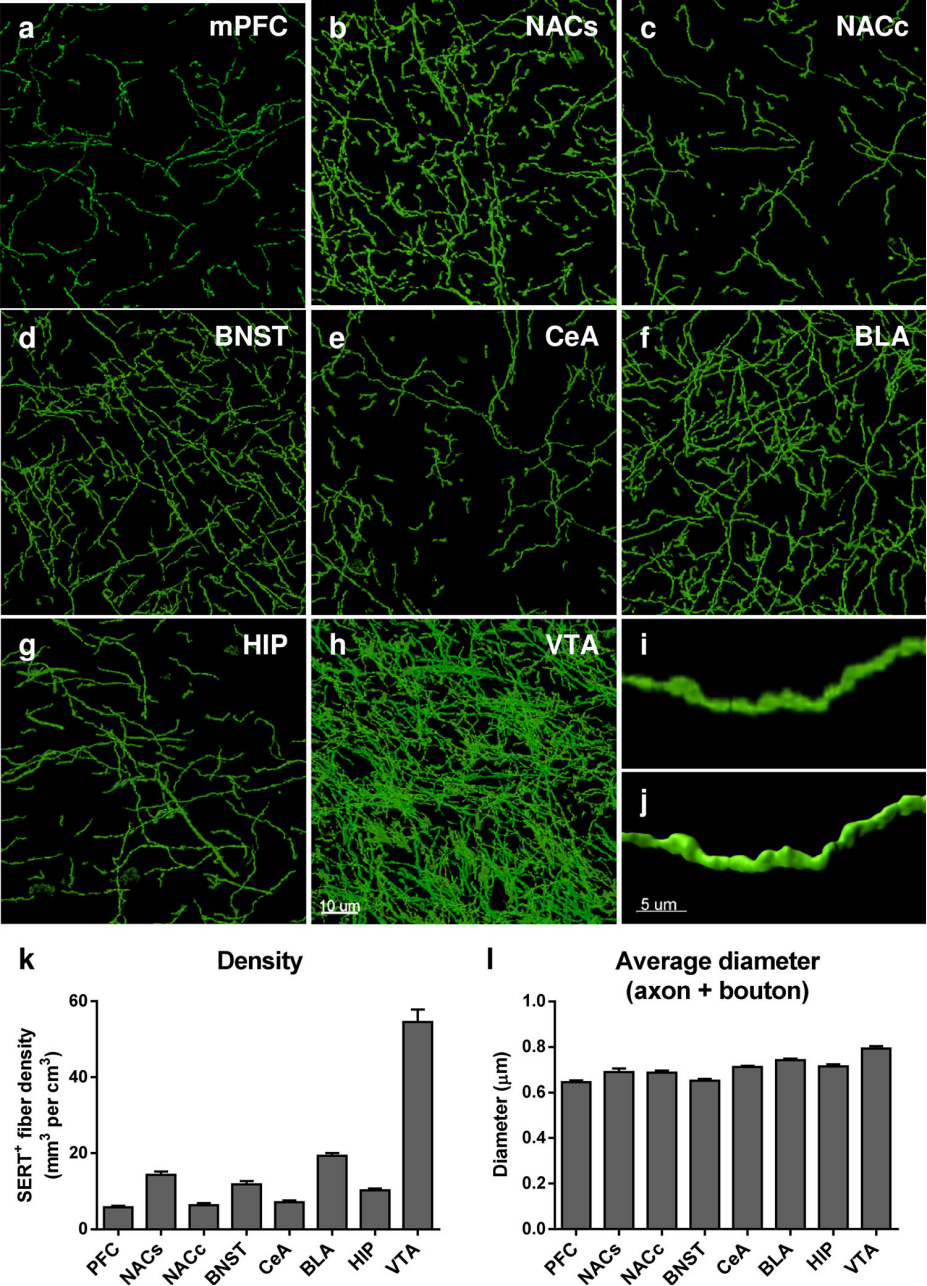
Distribution of SERT immunoreactive serotonergic axons throughout the limbic brain regions. **a**–**h** Representative 60x micrographs of SERT^+^ axons in the mPFC (**a**), NACs (**b**), NACc (**c**), BNST (**d**), CeA (**e**), BLA (**f**), HIP (**g**) and VTA (**h**). The micrographs correspond to the maximum intensity projection of 50 *z*-stacks across a 15-μm *z*-depth. **i** Higher magnification of a SERT immunoreactive axon in the BLA, and **j** shows the corresponding 3D reconstruction using Imaris software. **k** Quantification of the density of SERT immunoreactive fibers throughout the limbic brain. The results, expressed in mm^3^ of fibers per cm^3^ of tissue, are represented as the mean ± SEM of *n* = 30 images/brain region. **l** Extrapolated average diameter of SERT-immunoreactive fibers in each brain region (see ‘‘Material and methods’’). The results are expressed in μm and represented as the mean ± SEM of *n* = 30 images/brain region. *Scale bars*, **a**–**h** 10 μm; **i**–**j** 5 μm

**Fig. 3:**
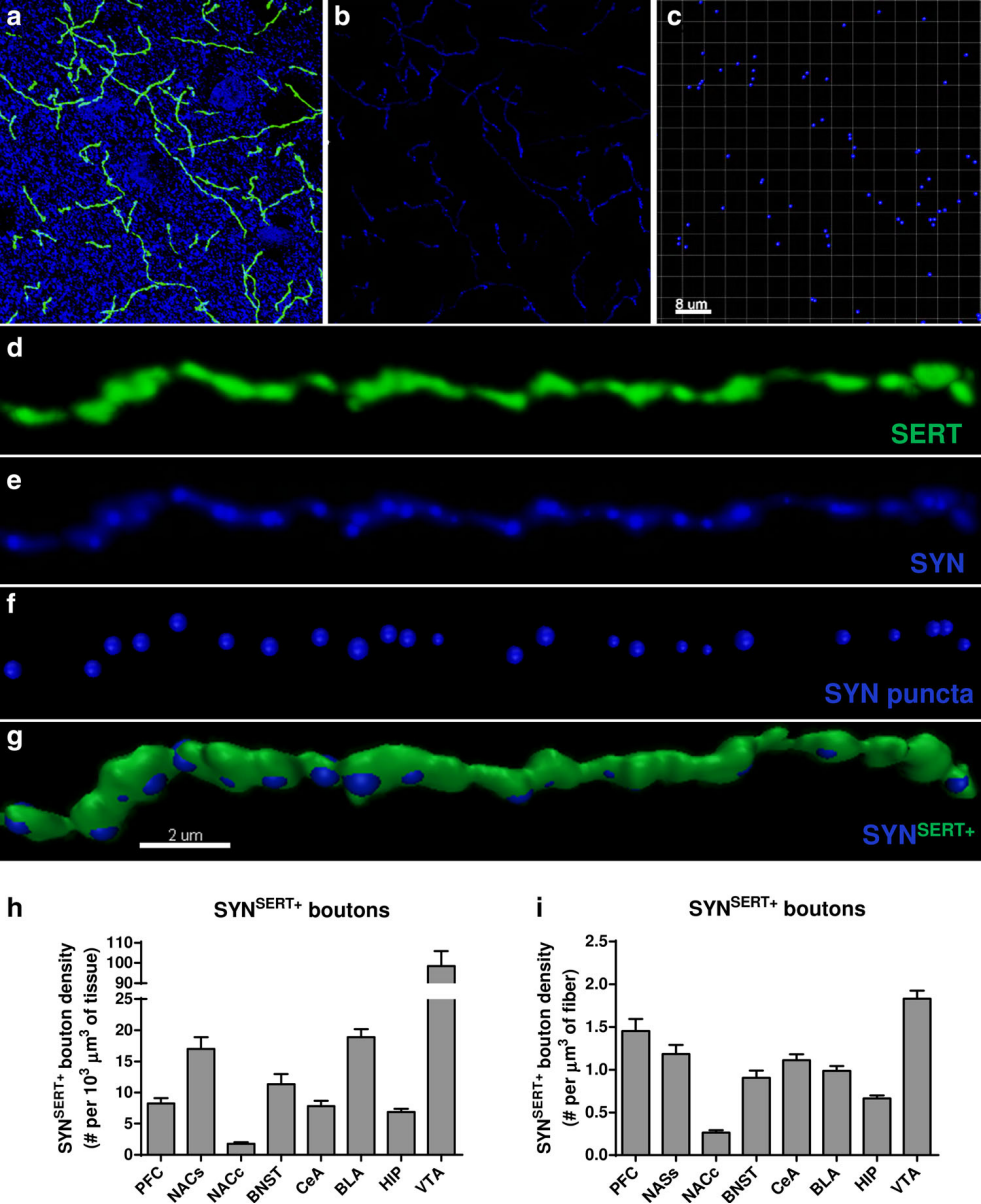
Identification and quantification of SYN^SERT+^ boutons within limbic brain areas. **a** Micrograph representing double immunostaining of SERT positive fibers (*green*) and the presynaptic marker synaptophysin (*blue*) in the mPFC. **b** Use of the masking function of Imaris software to mask the synaptophysin labeling located *outside* the SERT immunoreactive fibers. **c** Use of the *spot detection* function of Imaris software to identify the synaptophysin puncta with a minimum diameter of 0.4 μm. **d**–**g** Example of a 3D reconstruction of the synaptophysin immunoreactive boutons in SERT labeled fibers in the mPFC with Imaris software. **d** SERT-immunolabeled axon (*green*), **e** synaptophysin immunolabeling (*blue*) inside SER- immunolabeled fibers, **f** spot detection of synaptophysin puncta (*blue*) inside SERT-immunolabeled fibers, **g** 3D reconstruction of SERT-immunoreactive axons and the detected synoptophysin-positive boutons SYN^SERT+^. **h**–**i** volumetric quantification of the density of synaptophysin immunoreactive boutons within SERT-labeled fibers (SYN^SERT+^). The results are expressed in number of boutons per 10^3^ μm^3^ of tissue (**e**) or per μm^3^ of SERT-immunoreactive fibers (**f**) and represented as the mean ± SEM of *n* = 30 images/brain region. *Scale bars*, **a**–**c** 8 μm; **d**–**g** 2 μm

**Fig. 4:**
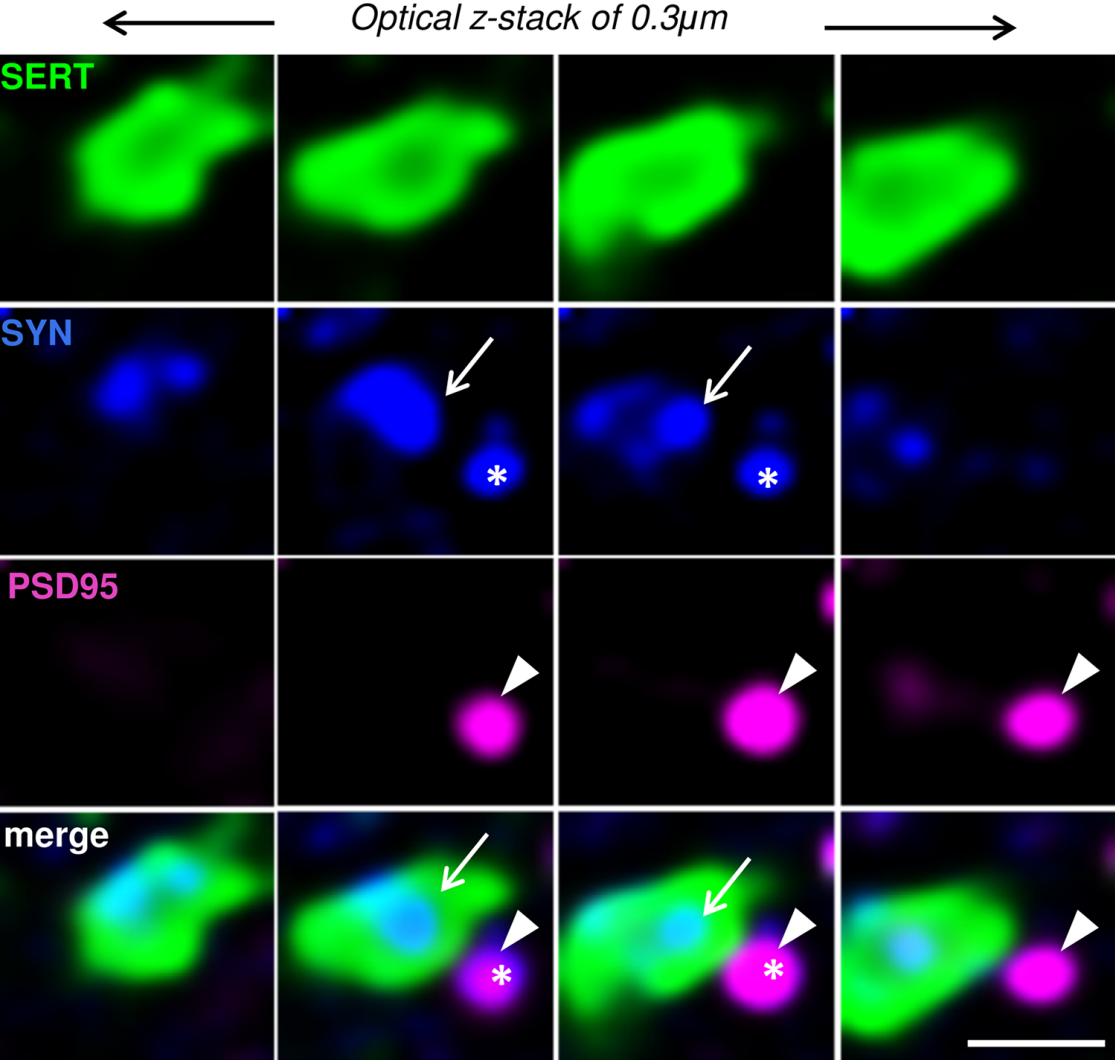
Example of the identification of a putative excitatory serotonergic triad. Serial *z*-stack micrographs of 0.3 μm *z*-step showing high-resolution images of SERT^+^ varicosities (*green*), the presynaptic marker synaptophysin (SYN, *blue*), and the postsynaptic marker PSD95 (*purple*) in the hippocampus. Synaptophysin labeling *inside* the SERT^+^ fibers (SYN^SERT+^ bouton, *arrow*) is in close apposition (0.6 μm) to a neurochemical excitatory synapse, defined by the close apposition (0.6 μm) to spot pairs of a synaptophysin (outside to SERT^+^ fibers, *star*) and PSD95 (*arrow head*). *Scale bar* 1 μm

**Fig. 5:**
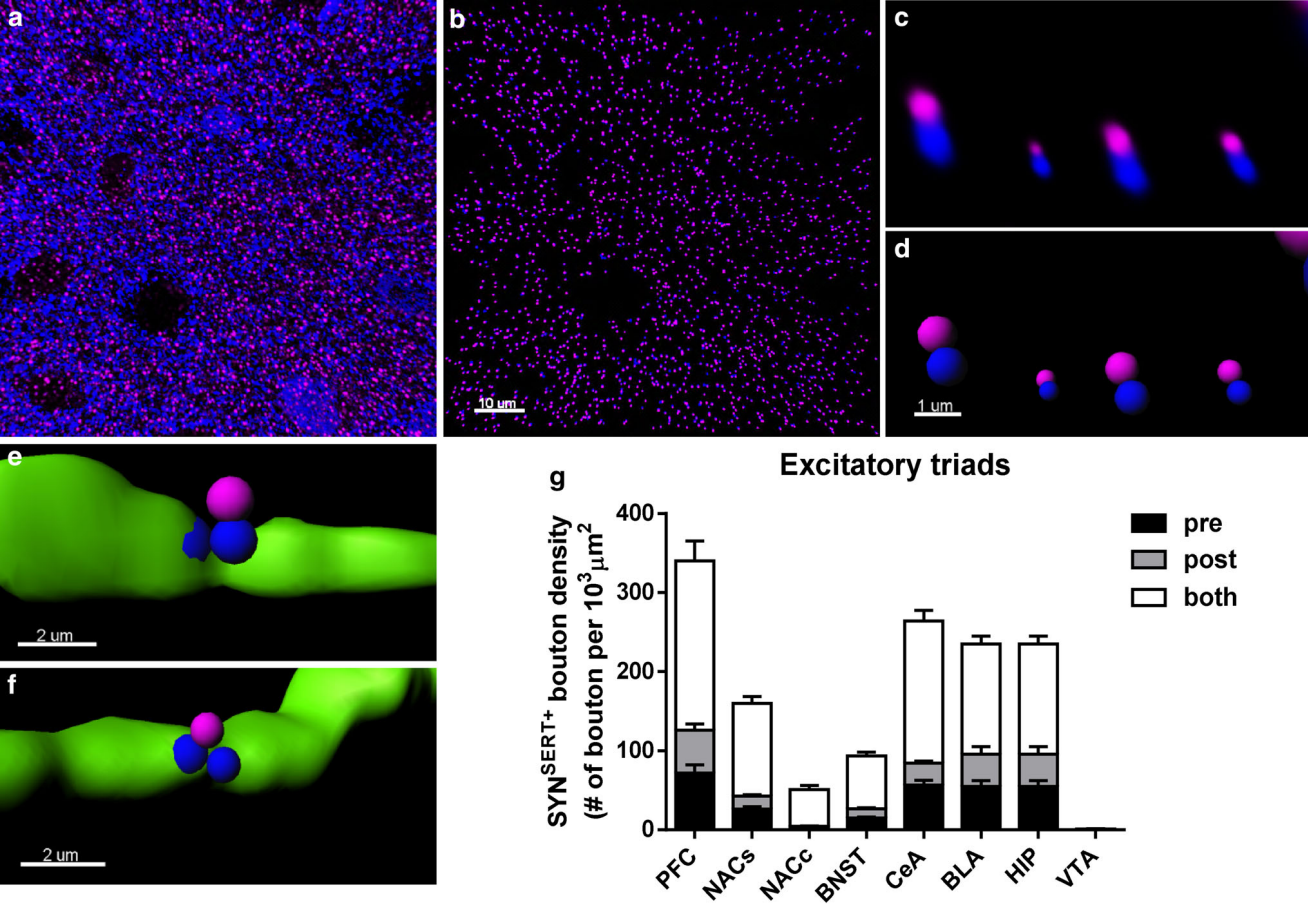
3D-reconstruction and quantification of excitatory 5-HTergic triads throughout the limbic brain. **a** Micrograph representing double immunostaining of the presynaptic marker synaptophysin (blue) and the excitatory postsynaptic marker PSD95 (purple) in the BLA. **b** The use of the *spot* *colocalization* function in Imaris to identify the synaptophysin (*blue*) and PSD95 (*purple*) spot pairs in the vicinity of 0.6 μm, which defined neurochemical excitatory synapses. **c** Higher magnification of synaptophysin (*blue*) and PSD95 (*purple*) spot pairs. **d** The use of the *spot detection* function of Imaris software to reconstruct the neurochemical excitatory synapses in 3D. **e** 3D reconstruction of SYN^SERT+^ boutons within SERT^+^ fibers (*green*) apposed to within 0.6 μm of the presynaptic specialization of a neurochemical excitatory synapse (*blue*). **f** 3D reconstruction of SYN^SERT+^ boutons in SERT^+^ fibers (*green*) orientated toward the postsynaptic specialization of a neurochemical excitatory synapse (*purple*). **g** Quantification of the density of putative excitatory triads. The results are expressed as the number of putative synapses per 10^3^ μm^3^ of SERT^+^ fiber. The densities of SYN^SERT+^ boutons preferentially orientated towards the presynaptic (*black*), postsynaptic (*dark gray*) or equidistant from both specializations (*light gray*) are represented as the mean ± SEM of *n* = 30 images/brain region. *Scale bars*, **a**, **b** 10 μm; **c**, **d** 1 μm; **e**, **f** 2 μm

Previous electron microscopy studies suggest that 5-HT is released from axonal varicosities that can be non-synaptic in close or relatively distant apposition to other dendrites and functions as ‘‘volume transmission” (Fig. 1a-1). In addition, 5-HT axon terminals engaged in asymmetric (Fig. 1a-2) or symmetric (Fig. 1a-3) synapses that directly contact the dendrites or axons of target neurons have also been observed. Studies have also shown that 5-HTergic boutons may synapse to either the pre- or postsynaptic components of GABA/glycinergic (Fig. 1a-4) or glutamatergic (Fig. 1a-5) synapses to form synaptic triads, which could have a modulatory effect on these synapses. By combining immunohistochemistry and 3D reconstruction, we mapped and quantified the distribution of putative 5-HT synaptic triads containing SYN^SERT+^ boutons located within 0.6 µm of neurochemical excitatory or inhibitory synapses (Fig. 1a, red boxes) in different limbic brain regions known to be densely innervated by 5-HTergic fibers. Our analysis included layers I–III of the prelimbic cortex (mPFC, bregma 2.1 ± 0.3 mm), the ventral nucleus accumbens core and the medial shell (NACc and NACs, bregma 1.18 ± 0.3 mm), the ventromedial and posterolateral part of the bed nucleus of the stria terminalis (BNST, bregma 0.18 ± 0.2 mm), the basolateral and central amygdala (BLA and CeA, bregma −1.46 ± 0.4 mm), the *strata oriens* of the CA3 region of the hippocampus (HIP, bregma −1.46 ± 0.4 mm) and the ventral tegmental area (VTA, bregma −2.92 ± 0.2 mm) (Fig. 1b).

We used well-validated, commercially available antibodies which are listed in Table 1, and the controls for antibody specificity along the two-stage immunohistochemistry protocol are provided in supplementary Figs. S1 and S2, respectively.

### Distribution of SERT-immunoreactive axons throughout the limbic brain

SERT-immunoreactive (SERT^+^) axons were reconstructed in 3D throughout eight brain regions using the surface-rendering tool of Imaris 8.1.2 software (Fig. 2a–j). We subsequently quantified their volumetric density in each limbic brain region here expressed as mm^3^ of fiber per cm^3^ of tissue (mm^3^/cm^3^) (Fig. 2k). We analyzed a total 195.43 mm of SERT^+^ fibers across eight brain regions, corresponding to a total volume of 821,456.07 μm^3^. We found an average SERT^+^ fiber density of 5.81 ± 0.34 mm^3^/cm^3^ in the mPFC, 14.33 ± 0.87 mm^3^/cm^3^ in the NACs, 6.32 ± 0.53 mm^3^/cm^3^ in the NACc, 11.81 ± 0.89 mm^3^/cm^3^ in the BNST, 19.32 ± 0.71 mm^3^/cm^3^ in the BLA, 7.12 ± 0.49 mm^3^/cm^3^ in the CeA, 10.24 ± 0.48 mm^3^/cm^3^ in the HIP and 54.54 ± 3.3 mm^3^/cm^3^ in the VTA (Fig. 2k). The highest SERT^+^ fiber density was found in the VTA followed by BLA > NACs > BNST > HIP > CeA > NACc > mPFC (Supp Table 1 for statistical analysis).

From the volume and the lateral surface of the reconstructed fibers obtained from Imaris software, we have calculated an average diameter of SERT immunoreactive axons throughout the sampled brain regions using the following formula: $D = \frac{4 \times V}{A}$, with *D*: diameter; *A*: lateral area and *V*: volume. This calculation gives an estimated average diameter taking the diameter of both the axon and the boutons into account. We found that SERT^+^ fibers have an average diameter (mean ± SEM) of 0.645 ± 0.007 μm in the mPFC, 0.689 ± 0.017 μm in the NACs, 0.687 ± 0.009 μm in the NACc, 0.652 ± 0.008 μm in the BNST, 0.742 ± 0.006 μm in the BLA, 0.712 ± 0.004 μm in the CeA, 0.715 ± 0.008 μm in the HIP and 0.793 ± 0.011 μm in the VTA (Fig. 2l). The highest SERT^+^ fiber diameter was found in the VTA > BLA > HIP > CeA > NACs > NACc > BNST > mPFC (Supp Table 2 for statistical analysis).

### Distribution of putative presynaptic boutons along SERT-positive axons (SYN^SERT+^)

To quantify the density of putative presynaptic boutons, we used the masking function in Imaris software to remove the labeling of the presynaptic marker synaptophysin (Syn^out^) that was located outside of the 3D-reconstructed SERT-immunoreactive fibers surface (Fig. 3a–e). We then performed a spot detection of the synaptophysin labeling (Fig. 3c, f) located inside the 3D-reconstructed SERT^+^ fibers (SYN^SERT+^) (Fig. 3g) and determined the volumetric density of SYN^SERT+^ presynaptic boutons per μm^3^ of tissue (Fig. 3h) or per μm^3^ of SERT-reconstructed fiber in each brain region (Fig. 3i). The average density of Syn^SERT+^ bouton per 10^3^ μm^3^ of tissue was (mean ± SEM) 8.26 ± 0.83 in the mPFC, 17.00 ± 1.88 in the NACs, 1.76 ± 0.24 in the NACc, 11.33 ± 1.65 in the BNST, 18.90 ± 1.29 in the BLA, 7.80 ± 0.85 in the CeA, 6.88 ± 0.48 in the HIP and 98.39 ± 7.40 in the VTA (Fig. 3h and Supp Table 3 for detailed statistical analysis), which corresponds to a density per μm^3^ of SERT^+^ fiber (mean ± SEM) of 1.45 ± 0.14 in the mPFC, 1.18 ± 0.10 in the NACs, 0.26 ± 0.02 in the NACc, 0.90 ± 0.08 in the BNST, 0.98 ± 0.05 in the BLA, 1.113 ± 0.06 in the CeA, 0.66 ± 0.03 in the HIP and 1.83 ± 0.09 in the VTA (Fig. 3i and Supp Table 4 for statistical analysis).

### Apposition of SYN^SERT+^ boutons to neurochemical excitatory synapses (“5-HT excitatory triads”)

By combining confocal fluorescence microscopy with Huygens deconvolution software, we generated high-resolution images to determine SERT^+^ boutons in close proximity to neurochemical excitatory synapses (Fig. 4). SERT^+^ boutons (SYN^SERT+^, Fig. 4, blue + green) were defined within the SERT^+^ varicosities (Fig. 4, green) using the presynaptic marker synaptophysin (Fig. 4, blue). Putative excitatory synapses (Fig. 4, blue + purple) were defined as spot pairs of PSD95 puncta (Fig. 4, purple) located within 0.6 μm of synaptophysin puncta located outside the SERT+ fibers (Fig. 4, blue).

To 3D reconstruct and quantify the density of SYN^SERT+^ boutons apposed to neurochemical excitatory synapses (Fig. 5a–g), we first masked the fluorescence signals of the excitatory pre- and postsynaptic markers located within the SERT^+^ fibers. This isolated the fluorescence signals of the excitatory pre- (Syn^out^) and postsynaptic markers (PSD95^out^) outside of the SERT^+^ fibers. Detection of Syn^out^/PSD95^out^ spot pairs within 0.6 μm allowed for quantification of neurochemical excitatory synapses in a manner similar to previous reports (Fogarty et al. 2013; Klenowski et al. 2015) (Fig. 5a–d). Then, we quantified the density of SYN^SERT+^ boutons that were located within 0.6 μm of the Syn^out^/PSD95^out^ spot pairs and determined whether they were located closer to either the presynaptic component (SYN) (Fig. 5e), the postsynaptic component (PSD95) (Fig. 5f) or both (equally distant) (Fig. 5g). The average density of excitatory triads expressed per 10^3^ μm^3^ SERT^+^ fiber was (mean + SEM) 340.70 ± 41.24 in the mPFC, 160.31 ± 11.09 in the NACs, 51.29 ± 5.40 in the NACs, 93.41 ± 7.19 in the BNST, 235.33 ± 23.06 in the BLA, 264.33 ± 19.97 in the CeA, 145.33 ± 12.40 in the HIP and 1.28 ± 0.34 in the VTA (Fig. 5g; Table 2 and Supp Table 5 for detailed statistical analysis). The majority of SYN^SERT+^ boutons were equidistant from the Syn^out^ and PSD95 markers (0.6 μm), and no significant differences in pre- or postsynaptic proximity were found in most of the brain regions analyzed. However, in the CeA, a higher proportion of SYN^SERT+^ boutons were located closer to the presynaptic component of the putative excitatory synapses, as compared with the postsynaptic component (CeA pre: 56 ± 6 vs CeA post: 28 ± 3 triads/10^3^ μm^3^ of SERT^+^ fiber, ***p = 0.0009, two-way ANOVA followed by Bonferroni post hoc analysis) (Table 2 and Supp Table 6 for statistical analysis).

**Table 2:** Density of SYN^SERT+^ synaptic triads

Brain region	Density of SYN^SERT+^ synaptic triads per 10^3^ μm^3^ of SERT^+^ fiber					
	Excitatory (mean ± SEM; *% ± SEM*)			Inhibitory (mean ± SEM; *% ± SEM*)		
	Pre	Post	Equidistant	Pre	Post	Equidistant
mPFC	72 ± 10	55 ± 8	214 ± 25	17 ± 3	7 ± 1	20 ± 4
	*20* ± *1*	*15* ± *1*	*65* ± *1.5*	*43* ± *5*	*17* ± *3*	*40* ± *3*
NACs	26 ± 3	16 ± 2	117 ± 8	61 ± 6*	29 ± 3	99 ± 10
	*16* ± *1*	*10* ± *1*	*74* ± *2*	*32* ± *1*	*15* ± *1*	*53* ± *1*
NACc	3.2 ± 0.7	1.3 ± 0.5	47 ± 5	2.5 ± 0.8	0.7 ± 0.5	3.3 ± 1
	*5* ± *1*	*2* ± *1*	*93* ± *2*	*50* ± *10*	*4* ± *3*	*46* ± *10*
BNST	15 ± 1.5	12 ± 1.5	66 ± 5	37 ± 6	18 ± 2.5	54 ± 6
	*15* ± *1*	*12* ± *1*	*73* ± *2*	*33* ± *2*	*17* ± *1.4*	*50* ± *2*
CeA	56 ± 6***	28 ± 3	180 ± 13	95 ± 10	69 ± 6	232 ± 21
	*20* ± *1.5*	*10* ± *1*	*70* ± *2*	*23* ± *1*	*18* ± *1*	*59* ± *1*
BLA	55 ± 7	40 ± 10	140 ± 10	90 ± 8	66 ± 5	217 ± 19
	*22* ± *1*	*15* ± *2*	*63* ± *2*	*24* ± *0.6*	*18* ± *0.5*	*58* ± *0.7*
HIP	33 ± 4	20 ± 2.5	92 ± 7.5	17 ± 2	17 ± 2	50 ± 5
	*21* ± *1*	*14* ± *1*	*65* ± *1*	*20* ± *1*	*20* ± *1.5*	*60* ± *2*
VTA	0.55 ± 0.19	0.07 ± 0.03	0.66 ± 0.15	160 ± 11	152 ± 10	529 ± 43
	*32* ± *6*	*6* ± *3*	*62* ± *6*	*19* ± *0.7*	*19* ± *0.7*	*62* ± *0.8*

Data in italic are expressed as mean and SEM in % of total excitatory or inhibitory triads

* *p* < 0.05; *** *p* < 0.001, as compared to SYN^SERT+^ boutons apposed to the postsynaptic component by two-way ANOVA analysis of variance followed by Bonferroni post hoc comparisons. An overall interaction was observed between both factors “brain region × pre/post/equidistant location of triads”, *F*(14, 464) = 62.87, *p* < 0.0001, with a main effect of each factor “brain region”, *F*(7, 232) = 75.32, *p* < 0.0001 and “pre/post/equidistant location of triads’’, *F*(2, 464) = 423.2, *p* < 0.0001

### Apposition of SYN^SERT+^ boutons to neurochemical inhibitory synapses (“5-HT inhibitory triads”)

**Fig. 6:**
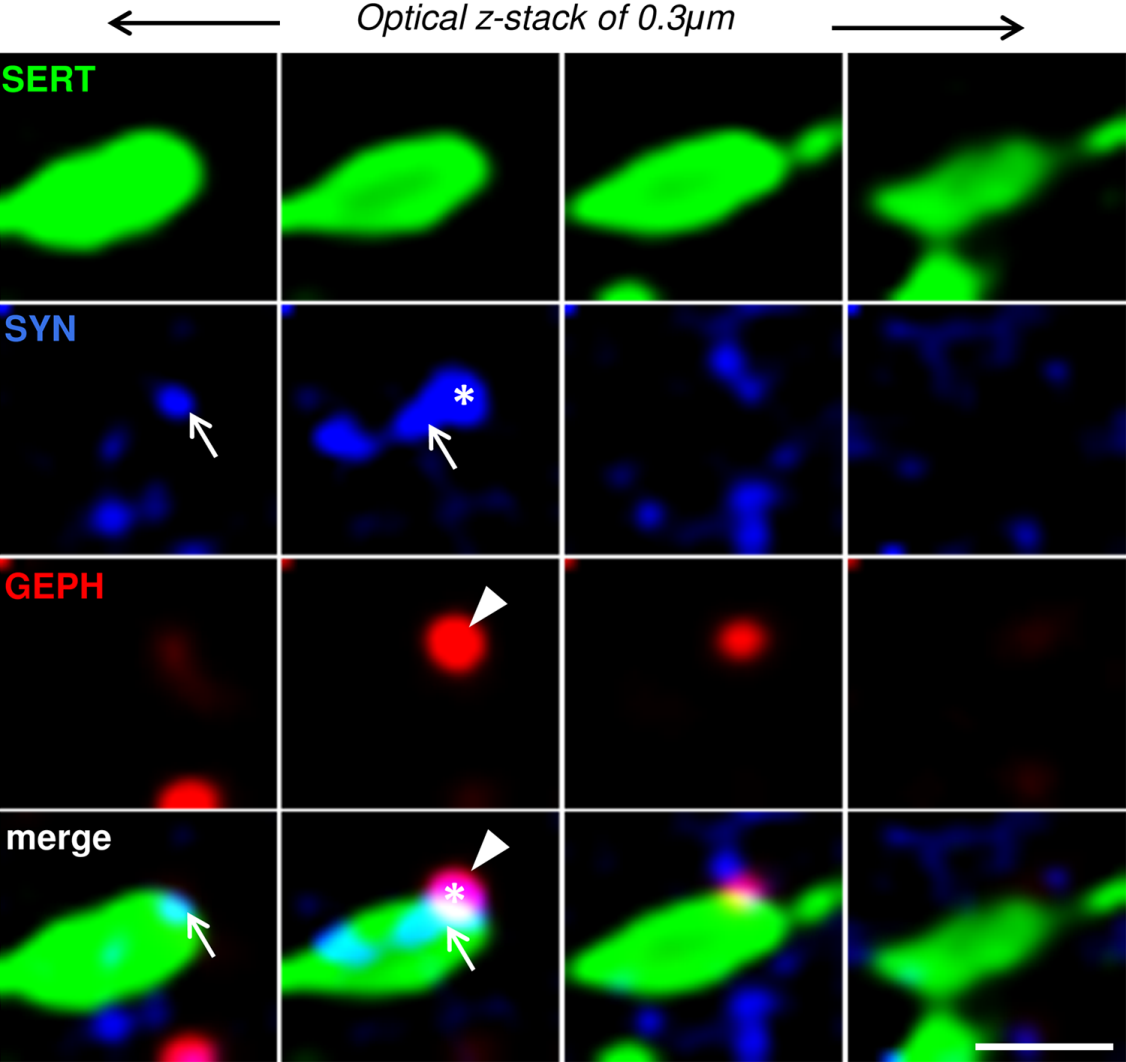
Example of the identification of a putative inhibitory serotonergic triad. Serial *z*-stack micrographs of 0.3-μm *z*-step showing high-resolution images of SERT^+^ varicosities (*green*), the presynaptic marker synaptophysin (SYN, *blue*), and the postsynaptic marker gephyrin (GEPH, *purple*) in the hippocampus. Synaptophysin labeling inside the SERT^+^ fibers (SYN^SERT+^ bouton, *arrow*) is in close apposition (0.6 μm) to a neurochemical inhibitory synapse, defined by the close apposition (0.6 μm) spot pairs of synaptophysin (outside to SERT^+^ fibers, *star*) and GEPH (*arrow head*). *Scale bar* 1 μm

We also used the abovementioned method to determine the density of SERT^+^ boutons in close proximity to neurochemical inhibitory synapses (Fig. 6). Similarly, putative SERT^+^ boutons (SYN^SERT+^, Fig. 6, blue + green) within the SERT^+^ varicosities (Fig. 6, green) were identified using the presynaptic marker synaptophysin (Fig. 6, blue), and putative inhibitory synapses (Fig. 6, blue + red) were identified as spot pairs of gephyrin (GEPH) puncta (Fig. 6, red) closely apposed to synaptophysin puncta outside the SERT^+^ fibers (Fig. 6, blue).

**Fig. 7:**
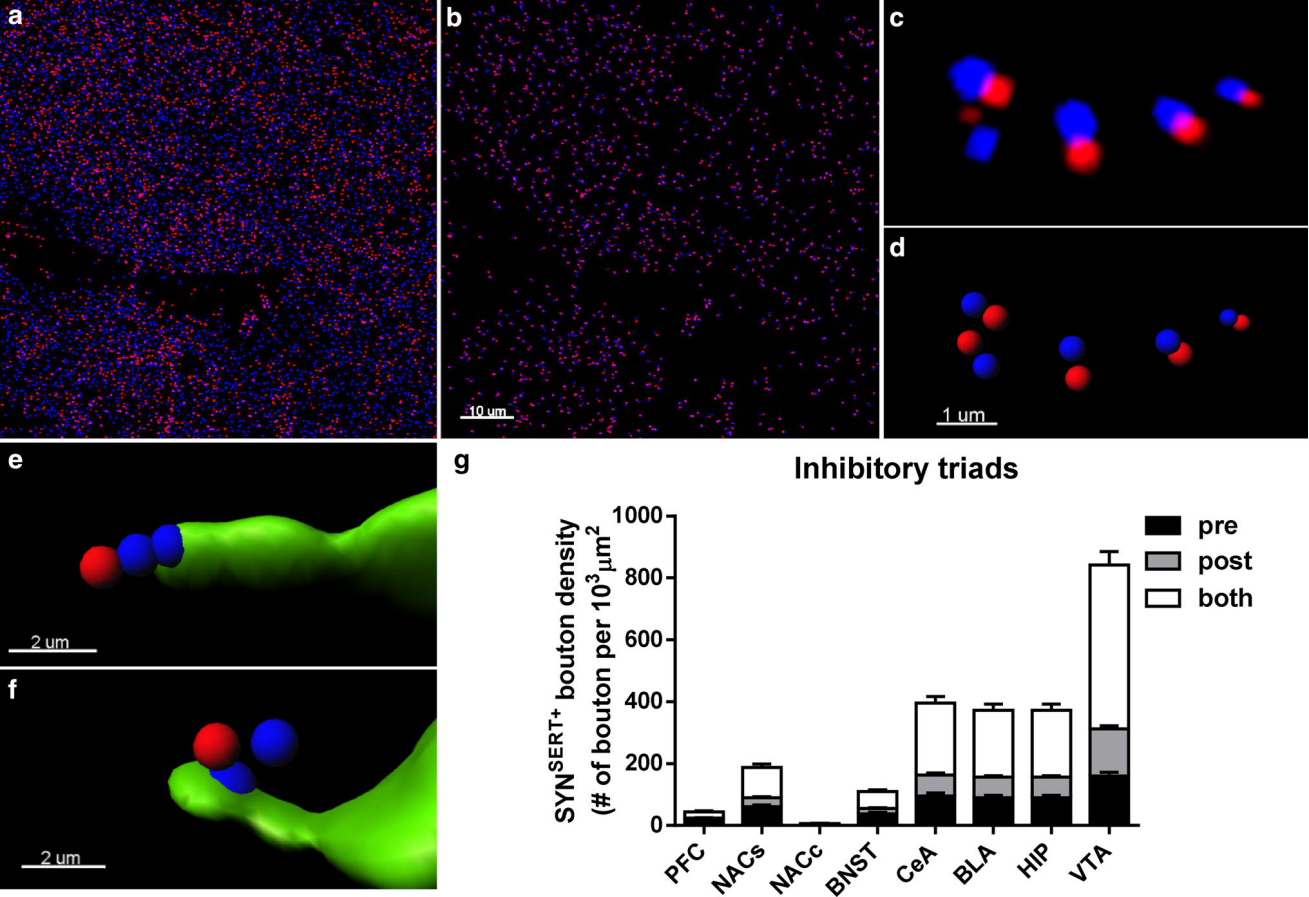
3D-reconstruction and quantification of inhibitory 5-HTergic triads throughout the limbic brain. **a** Micrograph representing a double immunostaining of the presynaptic marker synaptophysin (*blue*) and the inhibitory postsynaptic marker gephyrin (*red*) in the BLA. **b** Use of the *spot colocalization* function in Imaris to identify the synaptophysin (*blue*) and gephyrin (*red*) spot pairs in the vicinity of 0.6 μm, which defined neurochemical inhibitory synapses. **c** Higher magnification image of synaptophysin (*blue*) and gephyrin (*red*) spot pairs. **d** Use of the *spot detection* function of Imaris software to reconstruct the neurochemical inhibitory synapses in 3D. **e** 3D reconstruction of SYN^SERT+^ boutons within SERT+ fibers (*green*) apposed to within 0.6 μm presynaptic specialization of a neurochemical inhibitory synapse (*blue*). **f** 3D reconstruction of SYN^SERT+^ boutons in SERT+ fibers (*green*) orientated toward the postsynaptic specialization of a neurochemical inhibitory synapse (*red*). **g** Quantification of the density of putative inhibitory triads. The results are expressed as the number of putative synapses per 10^3^ μm^3^ of SERT^+^ fiber. The density of SYN^SERT+^ boutons preferentially orientated toward the presynaptic (*black*), postsynaptic (*dark gray*) or equidistant from both specializations (*light gray*) are represented as the mean ± SEM of *n* = 30 images/brain region. *Scale bars*, **a**, **b** 10 μm; **c**, **d** 1 μm; **e**, **f** 2 μm

We performed an identical spot analysis of the SYN^SERT+^ boutons in close proximity to neurochemical inhibitory synapses by quantifying the Syn^out^/Geph^out^ spot pairs (Fig. 7a–g). We found that the average density of inhibitory triads per 10^3^ μm^3^ of SERT^+^ fibers was (mean + SEM) 43.77 ± 7.27 in the mPFC, 188.33 ± 19.23 in the NACs, 6.47 ± 1.97 in the NACs, 109.37 ± 13.05 in the BNST, 372.77 ± 31.16 in the BLA, 396.03 ± 36.04 in the CeA, 84.64 ± 6.55 in the HIP, and 841.88 ± 62.48 in the VTA (Fig. 7g; Table 2 and Supp Table 7 for detailed statistical analysis). While no major difference was observed in most of the brain regions with respect to pre- and postsynaptic proximity, a greater proportion of SYN^SERT+^ boutons were closer to the presynaptic component of the putative inhibitory synapses in the NACs (NACs pre: 61 ± 6 vs NACs post: 29 ± 3 triads/10^3^ μm^3^ of SERT^+^ fiber, *p = 0.032, two-way ANOVA followed by Bonferroni post hoc analysis) (Table 2 and Supp Table 8 for statistical analysis).

### Proportion of total SYN^SERT+^ boutons engaged in triads

**Fig. 8:**
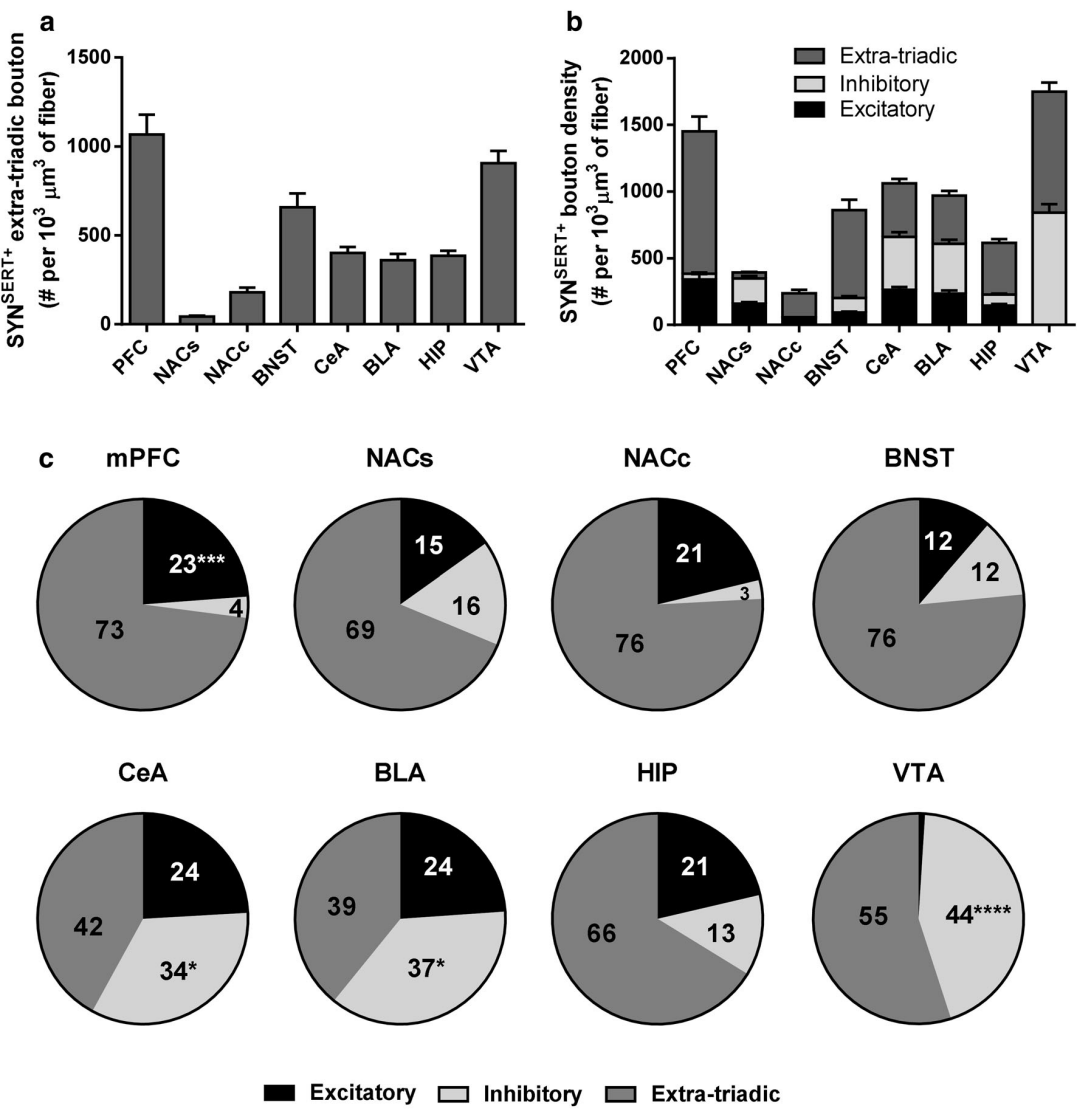
Quantification of extra-triadic, excitatory, and inhibitory triadic SYN^SERT+^ boutons throughout the limbic brain. **a** Quantification of extra-triadic SYN^SERT+^ boutons. The results are expressed as number of boutons per 10^3^ μm^3^ of SERT^+^ fiber and represented as the mean ± SEM of *n* = 30 images/brain region. **b** Quantification of extra-triadic, excitatory and inhibitory triadic SYN^SERT+^ boutons. The results are expressed as the number of boutons per 10^3^ μm^3^ of SERT^+^ fiber. The density of extra-triadic (*dark gray*), excitatory (*black*) or inhibitory (*light gray*) triadic boutons is represented as the mean ± SEM of *n* = 30 images/brain region. **c** Proportions of triadic vs extra-triadic boutons. The ratio of extra-triadic (*dark gray*), excitatory (*black*), and inhibitory (*light gray*) triadic boutons are represented as a percentage of the total density SYN^SERT+^ boutons for each brain region. *p < 0.05; ***p < 0.001 and ****p < 0.0001 from two-way ANOVA analysis of variance and Bonferroni post hoc comparison on the mean ± SEM calculated in (**b**)

To determine the proportion of total SYN^SERT+^ boutons engaged in putative excitatory/inhibitory triads across the eight brain regions (Fig. 8b–c), we used the total SYN^SERT+^ bouton density and the density of SYN^SERT+^ boutons forming synaptic triads, to calculate the density of SYN^SERT+^ boutons not involved in triadic contacts, here defined as extra-triadic boutons (Fig. 8a, Supp Table 9 for statistical analysis). We found that in the mPFC, the percentage of SYN^SERT+^ boutons forming synaptic triads was 27 % (117/434), which were mainly formed via close appositions to excitatory neurochemical synapses (100/434 = 23 %, ****p < 0.0001, two-way ANOVA followed by Bonferroni post hoc analysis). In the NACs, 31 % of SYN^SERT+^ boutons (280/900) formed triads which were distributed equally to excitatory (135/900 = 15 %) and inhibitory (145/900 = 16 %) neurochemical synapses. In the NACc, a higher, but not significantly different proportion of excitatory triads were found (18/93 = 21 %), as compared with inhibitory triads (2/93 = 3 %). Similarly, SYN^SERT+^ boutons forming triads in the BNST (140/600 = 24 %) were evenly distributed onto excitatory (68/600 = 12 %) and inhibitory (72/600 = 12 %) synapses. In the CeA and BLA, we observed a high proportion of SYN^SERT+^ boutons (251/438 = 58 % and 609/999 = 61 %, respectively) that were located closer to neurochemical inhibitory synapses (147/438 = 34 % and 368/999 = 37 %, *p = 0.0311 and *p = 0.0225, respectively, two-way ANOVA followed by Bonferroni post hoc analysis). The percentage of 5-HT excitatory synaptic triads was 24 % in both regions (104/438 and 241/999). In the HIP, the proportion of boutons forming triads was 33 % (121/365). The percentage SYN^SERT+^ boutons in proximity to putative excitatory or inhibitory synapses were 21 % (75/365) and 13 % (46/365) respectively. In the VTA, the SYN^SERT+^ boutons were preferentially located in close proximity to inhibitory neurochemical synapses (2291/5202 = 44 %, ****p < 0.0001, two-way ANOVA followed by Bonferroni post hoc analysis), and very few excitatory triads (4/5202 < 1 %) were observed (Fig. 8c and Supp Table 10 for statistical analysis).

Pearson correlation analysis showed no correlation between the ratios of excitatory/inhibitory triads along SERT^+^ fibers and the ratios of putative excitatory/inhibitory synapses throughout the eight brain regions analyzed (Pearson *r* = 0.5744; number of XY pairs = 8; *p* = 0.1365, Supp Fig. 4), which suggests that the volumetric density of SYN^SERT+^ boutons forming excitatory or inhibitory triads is not driven by the abundance of neurochemical excitatory or inhibitory synapses within each brain region.

## Discussion

In this study, we have adapted a semi-automated method combining multi-step immunolabeling and high-resolution confocal imaging to reconstruct in 3D a total of 195.43 mm of SERT-immunoreactive axons and map the distribution of synaptophysin-immunoreactive boutons forming synaptic triads with excitatory or inhibitory neurochemical synapses throughout the mouse limbic brain. For the first time, we provide a comparative volumetric quantification of the distribution SERT-immunoreactive axons and the density of serotonergic excitatory and inhibitory synaptic triads within several limbic areas of the mouse brain.

### Use of the serotonin transporter SERT as a marker of serotonergic axons

Serotonin immunolabeling has been extensively used to label serotonergic axons and quantify their densities in the brain. However, serotonin is a neurotransmitter which can be rapidly metabolized, and thus, the use of antibodies to label serotonin might underestimate the density of serotonin axons. Here, we choose to use an antibody against the serotonin transporter, which has been shown to be a more robust marker of serotonergic axons than 5-HT itself (Nielsen et al. 2006). However, precautions should also be considered when using SERT as a marker of 5-HT neurons due to the subtle differences that may be detected between SERT and 5-HT immunolabeling. During development, some non-5-HT producing neurons in the thalamus, limbic cortex, hypothalamus, retina and superior olivary nucleus transiently express the SERT (Lebrand et al. 1998; Narboux-Nême et al. 2008). In adult rats, although comparable labeling has been observed throughout the cerebral cortex and striatum with only minor differences in the hippocampus, entorhinal cortex and the NAC core, more pronounced differences have been detected in the caudal part of the NAC shell (Brown and Molliver 2000). This previous work has shown that the NAC shell is innervated by two functionally different types of 5-HTergic axons that either contains or lacks the SERT. Our method of labeling of 5-HT fibers with an antibody directed against SERT may have only uncovered a particular subset of serotonergic axons expressing SERT in the NAC shell and is, therefore, likely to have underestimated the density of serotonergic axons and their triadic associations in this region. Further work is needed to determine the distribution and proportion of 5-HT axons lacking the SERT in the mouse NAC shell as well as in other limbic brain regions.

### Serotonergic triads, electron microscopy, and physiology

A similar approach to the one used in our study combined array tomography and high-resolution immunolabeling to identify 5-HT synaptic triads in the dorsal raphe nuclei. Both glutamatergic and GABAergic terminals converged onto serotonergic TPH-labeled neurons to modulate of the excitatory activity of serotonergic neurons in rats (Soiza-Reilly et al. 2013; Soiza-Reilly and Commons 2014). We therefore focused on mapping SERT-immunoreactive axons fiber density and distribution of putative serotonergic boutons forming synaptic triads with excitatory or inhibitory neurochemical synapses in mouse limbic brain regions known to receive a high level of 5-HT synaptic input. Our comprehensive assessment and detailed statistical analysis of the distribution of “excitatory vs inhibitory” serotonergic triads and their “pre- vs postsynaptic” location have highlighted some specific differences in the architecture of serotonergic triads within limbic brain areas. These data suggest that 5-HT axons might differentially modulate excitatory or inhibitory transmission in these brain regions, however, further work is needed to confirm the functional significance and ultrastructural distribution of these triads throughout the mouse brain.

In the layer I–III of the prefrontal cortex, the most densely 5-HT-innervated layer in the cortex (Audet et al. 1989), we showed that SYN^SERT+^ boutons preferentially formed triads with neurochemical excitatory synapses, suggesting that 5-HT may have a preferential role in the regulation of glutamate transmission in this region. In line with this, electron microscopy studies have observed that junctional and non-junctional appositions of 5-HT-immunoreactive axons to non-5-HTergic axons or dendrites engaged in asymmetrical (excitatory) synapses in a triadic formation were frequently encountered in the upper layers of the frontal cortex in rats (Séguéla et al. 1989), suggesting both a pre- or postsynaptic control of excitatory transmission in this brain region. Electrophysiology studies in rats have also shown 5-HT-mediated pre- and postsynaptic effects in this brain region including increases both glutamate release and the amplitude of glutamatergic postsynaptic currents (EPSCs) (Aghajanian and Marek 1997). These interactions between 5-HT and glutamate within functional triadic contacts could play an important role in the etiology of psychosis and be a promising target for the development of antipsychotics (Marek and Aghajanian 1998) and the treatment of schizophrenia (González-Maeso et al. 2008).

In the NAC core and shell, we show that SYN^SERT+^ boutons were equally distributed among excitatory and inhibitory neurochemical synapses with a non-significant preference to the formation of excitatory triads in the NACc. Triadic associations of 5-HT- or SERT-immunolabelled axons to non-serotonergic axons forming both symmetrical (inhibitory) or asymmetrical (excitatory) synapses have been previously observed by electron microscopy in the NAC core and shell of rats (Van Bockstaele and Pickel 1993; Pickel and Chan 1999). The proximity of 5-HT-positive axon terminals to GABA terminals engaged in symmetrical (inhibitory) synapses revealed by electron microscopy in rats (Van Bockstaele et al. 1996) suggests that 5-HT could influence the release of GABA. This is supported by functional studies showing a modulation of GABA release in the NAC by 5-HT2C receptors in rats (Kasper et al. 2015). Interestingly, we found a significantly higher proportion of SYN^SERT+^ boutons located closer to the presynaptic component of putative inhibitory synapses within the NAC shell, suggesting that 5-HT could have a modulatory effect on GABA release from GABAergic synapses in this area. Furthermore, functional studies have also revealed 5-HT-mediated control of glutamate release in rat NAC core and shell slices via activation of presynaptic 5-HT1B receptors (Muramatsu et al. 1998), however, we did not observe any preferential location of SYN^SERT+^ boutons to the pre- or the postsynaptic component of excitatory triads. Whether 5-HTergic boutons are truly equally distributed toward the pre- and postsynaptic components of glutamate synapses to regulate glutamate signaling in the mouse NAC or whether our methodology using a SERT antibody only focused on a particular subtype of 5-HT neurons in this region remains to be determined.

Among the brain regions analyzed, we found that the highest proportions of SYN^SERT+^ boutons engaged in triads were in the BLA and CeA of the amygdala, reaching 61 and 58 %, respectively. We observed a slightly higher number of SYN^SERT+^ boutons located in closer apposition toward inhibitory neurochemical synapses than excitatory synapses. In the BLA, serotonergic triads apposed to both symmetrical (inhibitory) and asymmetrical (excitatory) synapses have been identified by electron microscopy in rats (Muller et al. 2007). We have previously shown that in the rat BLA, interneurons contain a significantly higher neurochemical GABAergic synapse density compared with principal neurons (Klenowski et al. 2015). The preferential proximity of SYN^SERT+^ boutons to neurochemical inhibitory synapses could therefore suggest that serotonergic axons projecting to the BLA may preferentially target local interneurons and modulate their activity. Although this requires further investigation, previous electrophysiology reports have demonstrated preferential 5-HT mediated effects on interneurons in the BLA (Rainnie 1999). At a presynaptic level, GABA release in rat BLA slices was shown to be inhibited by 5-HT1A (Koyama et al. 1999, 2002; Kishimoto et al. 2000) and activated by 5-HT2A (Jiang et al. 2008) and 5-HT3 (Koyama et al. 2000, 2002) receptors. Combined these results are consistent with 5-HT connectivity structured toward the regulation of inhibitory GABAergic activity in the BLA (Muller et al. 2007). Because the excitability of BLA principal cells, which presumably represents a functional basis of anxiety states, is potently controlled by local GABAergic interneurons (Rainnie et al. 1991; Washburn and Moises 1992; Lang and Paré 1997, 1998), the regulation of GABAergic synapses by 5-HT is in line with a potential role of 5-HT signaling in the BLA in anxiety-related behaviors (Strauss et al. 2013; Vicente and Zangrossi 2014).

The CeA consists primarily of GABAergic projection neurons and interneurons (Sun and Cassell 1993; Veinante and Freund-Mercier 1998) that integrate and modulate glutamatergic inputs from the thalamus, cortex and BLA (LeDoux 2007) to mediate behavioral and physiological responses associated with fear/anxiety (Kalin et al. 2004; Ciocchi et al. 2010) and various negative emotional states including stress/anxiety following alcohol withdrawal (Roberto et al. 2012; Gilpin et al. 2015). Our results showing the preferential locality of SYN^SERT+^ boutons to inhibitory synapses in the CeA are consistent with the contribution of 5-HT signaling in the regulation of anxiety-related behaviors (Mo et al. 2008) being facilitated by modulation of the local GABAergic microcircuit in the CeA (Ciocchi et al. 2010; Jiang et al. 2014). Further functional studies are however needed to confirm the role of 5-HT in the regulation of local GABAergic circuitry.

In the VTA, we observed the highest density of SERT^+^ fibers and SYN^SERT+^ boutons, as well as the largest average fiber diameter. This result was likely given the proximity of the VTA to the raphe nucleus. Importantly, almost half of these total boutons (44 %) were located in close proximity to putative inhibitory synapses and a very low number (<1 %) in the proximity of putative excitatory synapses. Considering the large network of GABAergic neurons in the VTA, this result was somewhat expected and is in line with previous electron microscopy (Hervé et al. 1987) and functional studies. For example, 5-HT1B agonist application was shown to reduce [3H]-GABA release (Johnson et al. 1992; Yan and Yan 2001) and GABA_B_-mediated IPSCs in VTA dopamine (DA) neurons (Cameron and Williams 1994). Furthermore, cocaine-induced reductions in GABA_B_ inhibitory postsynaptic potentials in DA neurons of rat VTA slices were found to be mediated by 5-HT1B receptor activation (Cameron and Williams 1994), which, in turn, facilitates cocaine-induced increases in DA levels in the NACc (Parsons et al. 1999; O’Dell and Parsons 2004). Collectively, these data suggest that 5-HT modulation of VTA signaling occurs primarily via the regulation of local and/or non-local inhibitory synapses. Dopaminergic neurons in the VTA that project to the NAC to form the mesolimbic reward pathway are sensitive to 5-HT/GABA interactions which affect the release of DA in the NAC, notably, in response to cocaine (Cameron and Williams 1994; O’Dell and Parsons 2004), MDMA (Bankson and Yamamoto 2004) and alcohol (Theile et al. 2009). Our methodology could help to identify changes in SERT^+^ fiber density and 5-HT connectivity contributing to the dysregulation of DAergic signaling that is associated with the development of addictive behaviors.

### Methodological considerations

Electron microscopy studies have revealed that 5-HT axons directly contact the dendrites and the cell bodies of various types of neurons via symmetrical or asymmetrical synapses, and also form triadic contacts with dendrites or axons that are engaged in synapses (for review, see Descarries et al. 2010). Symmetrical and asymmetrical synapses have been proposed to be inhibitory and excitatory, respectively; however, specific markers for the postsynaptic component of 5-HT symmetrical or asymmetrical synapses identified from EM have not yet been determined. Consequently, the quantitative distribution of these synapses cannot be conclusively resolved by fluorescence microscopy. Therefore, our study focused on quantifying the density of serotonergic boutons in close proximity to “conventional” synapses, identified using well-validated neurochemical markers of excitatory and inhibitory pre- and postsynaptic specializations, to estimate the density and distribution of serotonergic excitatory/inhibitory triads throughout the mouse limbic brain.

Interestingly, in some brain regions, including the mPFC, NACc, HIP, and VTA, the proportion of excitatory, inhibitory, and extra-triadic SYN^SERT+^ boutons that we observed were in line with the proportion of asymmetrical, symmetrical, and extra-synaptic 5-HT boutons observed in previous electron microscopy studies (Hervé et al. 1987; Séguéla et al. 1989; Oleskevich et al. 1991; Van Bockstaele and Pickel 1993; Smiley and Goldman-Rakic 1996; Miner et al. 2000). If direct asymmetrical and symmetrical synapses made by 5-HT boutons on dendrites are presumed to be excitatory and inhibitory, respectively, our data suggest that the excitatory/inhibitory balance of 5-HT bouton connectivity is a common structural feature related to both 5-HTergic direct synapses and synaptic triads in brain regions that we have investigated. However, additional EM studies that characterize the postsynaptic densities of asymmetrical and symmetrical 5-HT synapses are required to verify this hypothesis.

The methodology described here is highly dependent on the specificity of the antibodies used. Careful consideration should be given to the choice of antibodies and the control of the non-specific labeling of each antibody, to determine the right antibody dilution, labeling sequence and to ensure the best signal-to-noise ratio. Here, we used a combination of specific, well-validated antibodies to achieve high-quality immunolabeling and high-resolution imaging. Future investigations implementing this methodology should also be aware that the generation of consistent quantitative data is highly dependent on slice preparation.

In summary, this method allows for a fast quantitative analysis of 5-HT innervation and connectivity in the mouse brain. The combination of this technique with other methods, for example, with the reconstruction of neurobiotin-filled neurons (Fogarty et al. 2013; Klenowski et al. 2015), offers the possibility for the distribution of 5-HTergic excitatory/inhibitory triads along the dendritic trees, axon or soma of a single intracellularly labeled neuron to be determined in the future studies.

## Conclusion

Serotonin neurons are highly plastic both during development and in the mature brain (Azmitia 1999). Furthermore, alterations in 5-HT signaling have been implicated in the etiology of various neuropsychiatric disorders, including stress, anxiety, depression, and addiction. For example, changes in serotonin neuronal innervation and function have been observed in rodents or monkeys following prenatal exposure to stress (Miyagawa et al. 2011), alcohol (for review, see Belmer et al. 2016) or cocaine (Snyder-Keller and Keller 1993). Early life or adulthood exposure to stress (Kuramochi and Nakamura 2009; Xue et al. 2013; Ohta et al. 2014), MDMA (Hatzidimitriou et al. 1999) or selective serotonin reuptake inhibitors (Zhou et al. 2006) also facilitate changes in 5-HTergic signaling. This high-throughput screening method provides a valuable tool for determining how 5-HTergic neuron plasticity is modulated following chronic exposure to anxious stimuli, stressors or drugs of abuse which can be investigated using behavioral paradigms in rodents. Furthermore, given the fact that transgenic mouse lines are now available to identify axonal projections of many neuronal types, such as cholinergic (Tallini et al. 2006), dopaminergic (Zhou et al. 2009), and noradrenergic (Kanazawa et al. 2010) neurons, this method could be adapted to map the changes in the plasticity of several neuronal pathways. A detailed analysis of the functional connectivity of these neuronal populations will help to provide a greater understanding of how brain connectivity is altered in numerous mental and psychiatric disorders.

## Electronic supplementary material

Below is the link to the electronic supplementary material.

Supplementary material 1 (TIFF 7852 kb)

Supplementary material 2 (TIFF 6667 kb)

Supplementary material 3 (TIFF 6110 kb)

Supplementary material 4 (TIFF 153 kb)

Supplementary material 5 (DOCX 15 kb)

Supplementary material 6 (DOCX 73 kb)
