# Supplementary figures and images for: Mapping the connectivity of serotonin transporter immunoreactive axons to excitatory and inhibitory neurochemical synapses in the mouse limbic brain
Source: Brain Struct Funct. 2016 Aug 2;222(3):1297–314. doi: 10.1007/s00429-016-1278-x (PMC5368196; doi:10.1007/s00429-016-1278-x)

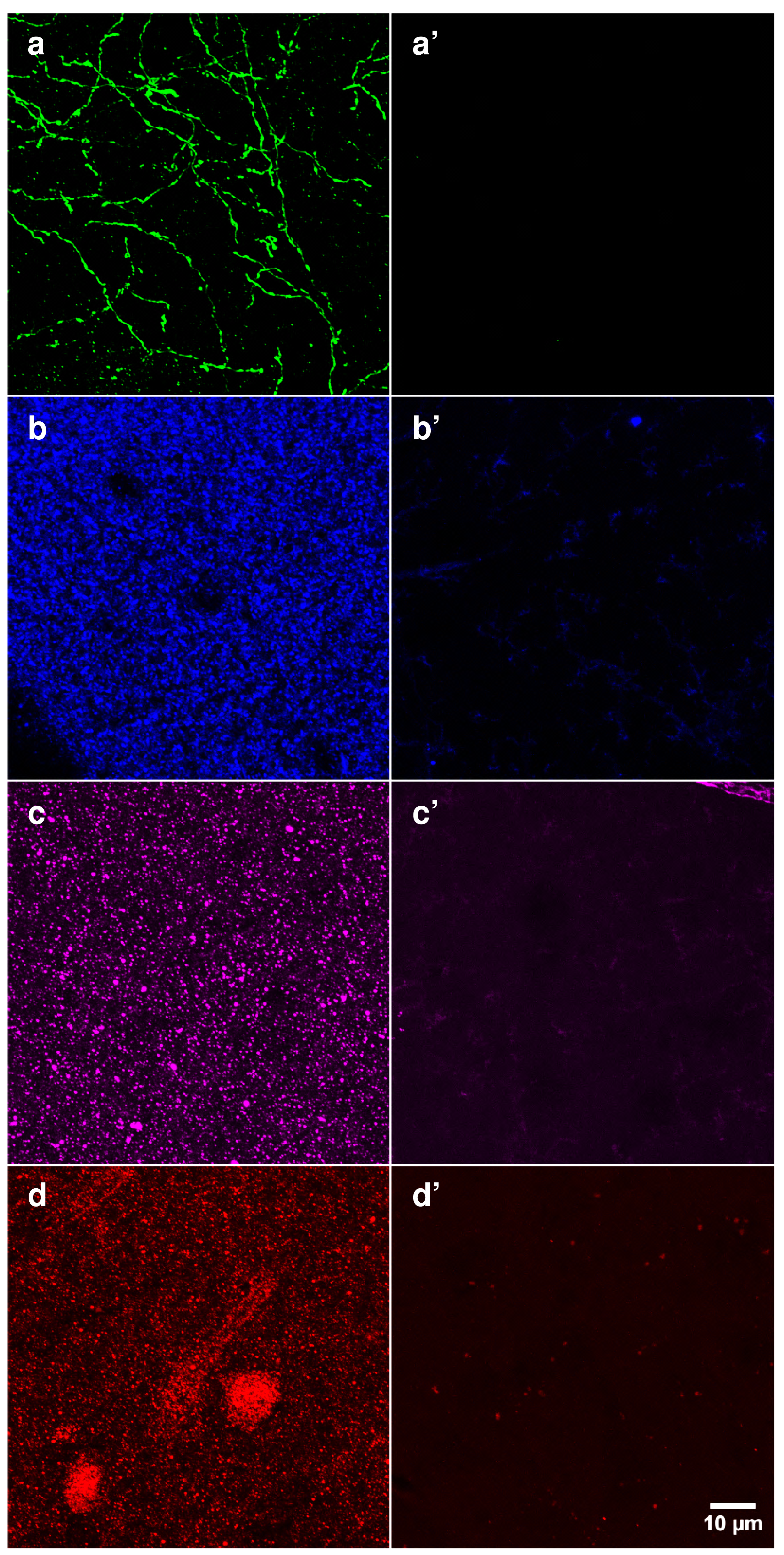

Supplement: Supplementary file 1 — Supplementary material 1 (TIFF 7852 kb) [file 429_2016_1278_MOESM1_ESM.tif]

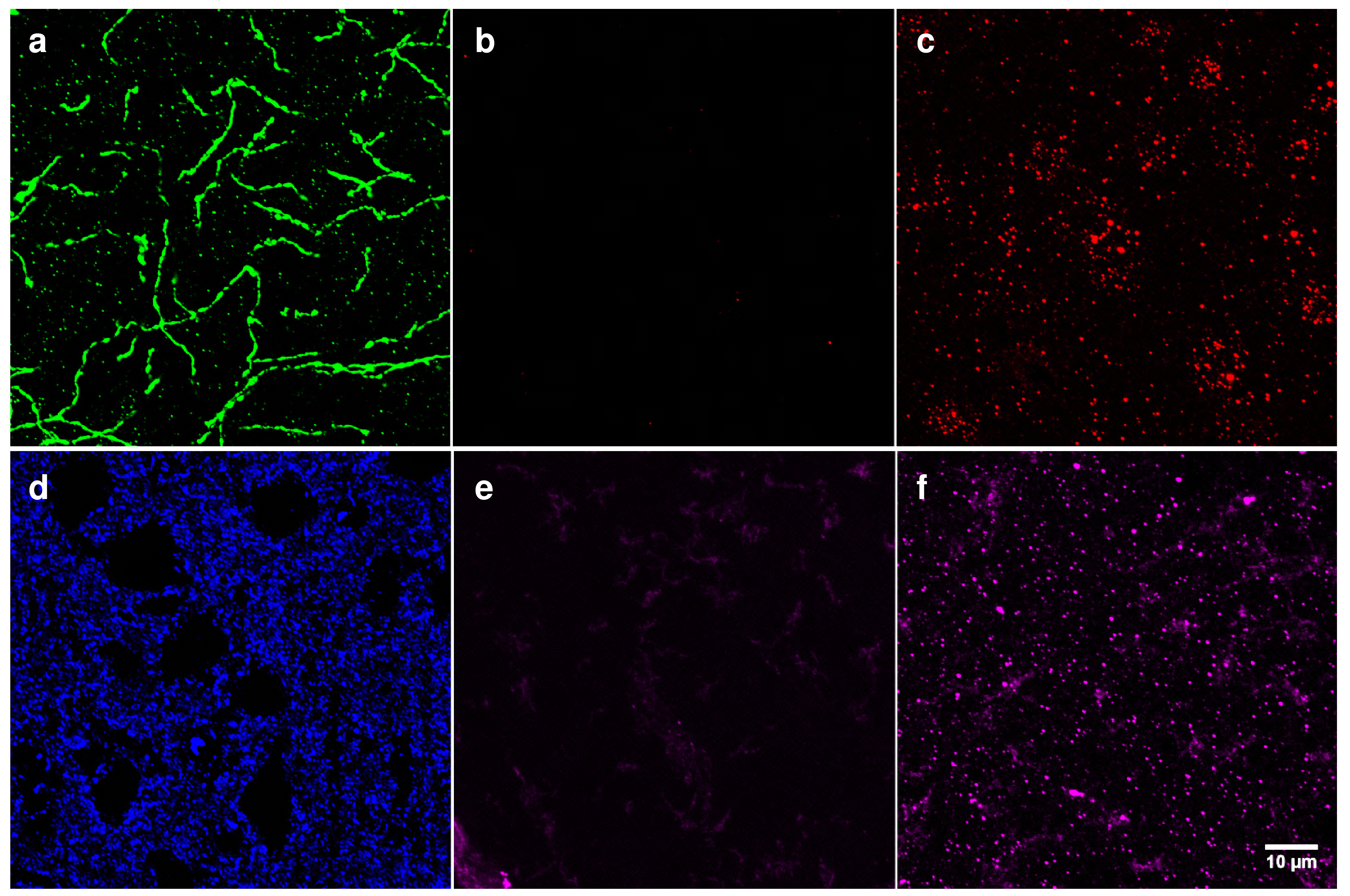

Supplement: Supplementary file 2 — Supplementary material 2 (TIFF 6667 kb) [file 429_2016_1278_MOESM2_ESM.tif]

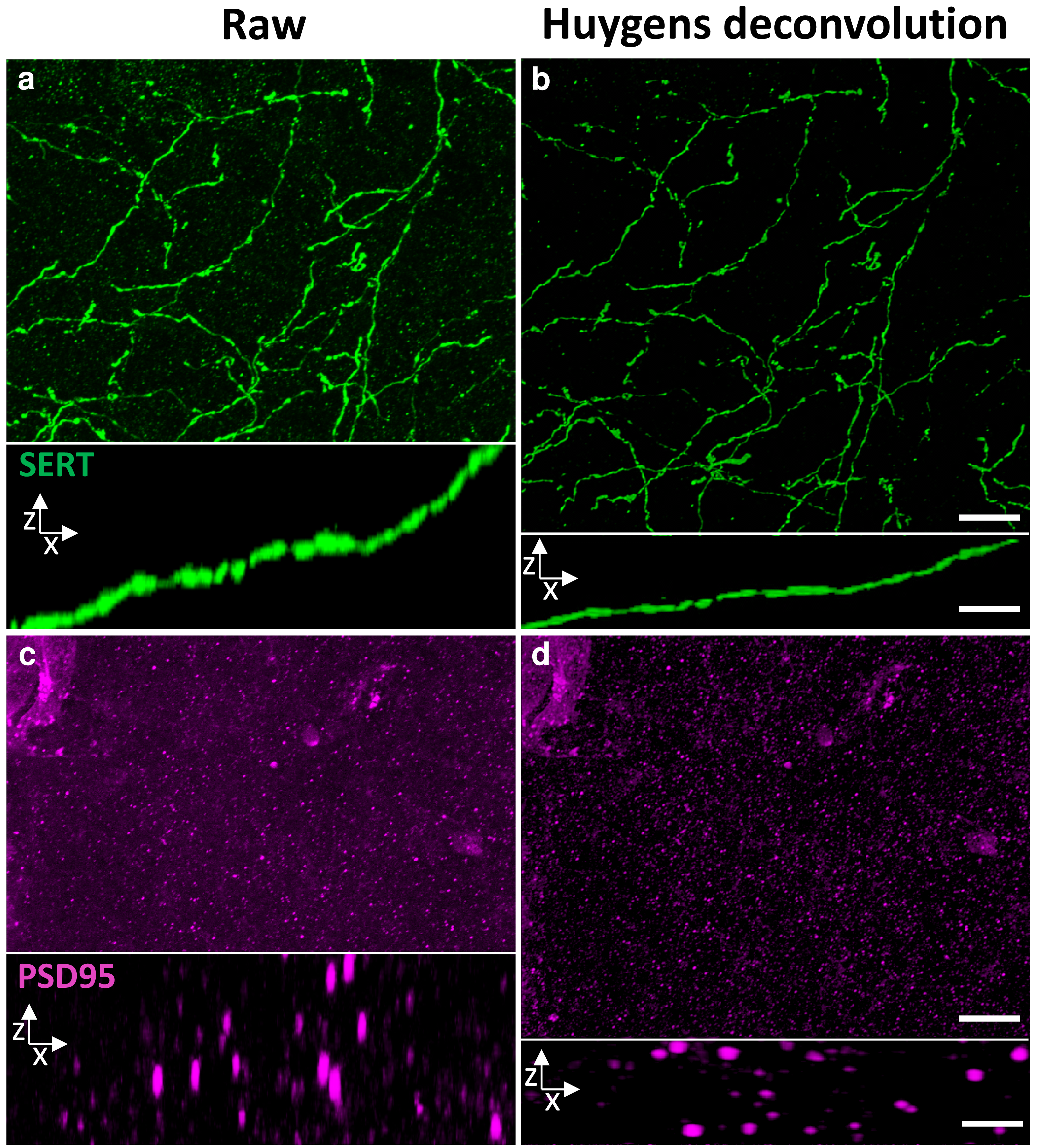

Supplement: Supplementary file 3 — Supplementary material 3 (TIFF 6110 kb) [file 429_2016_1278_MOESM3_ESM.tif]

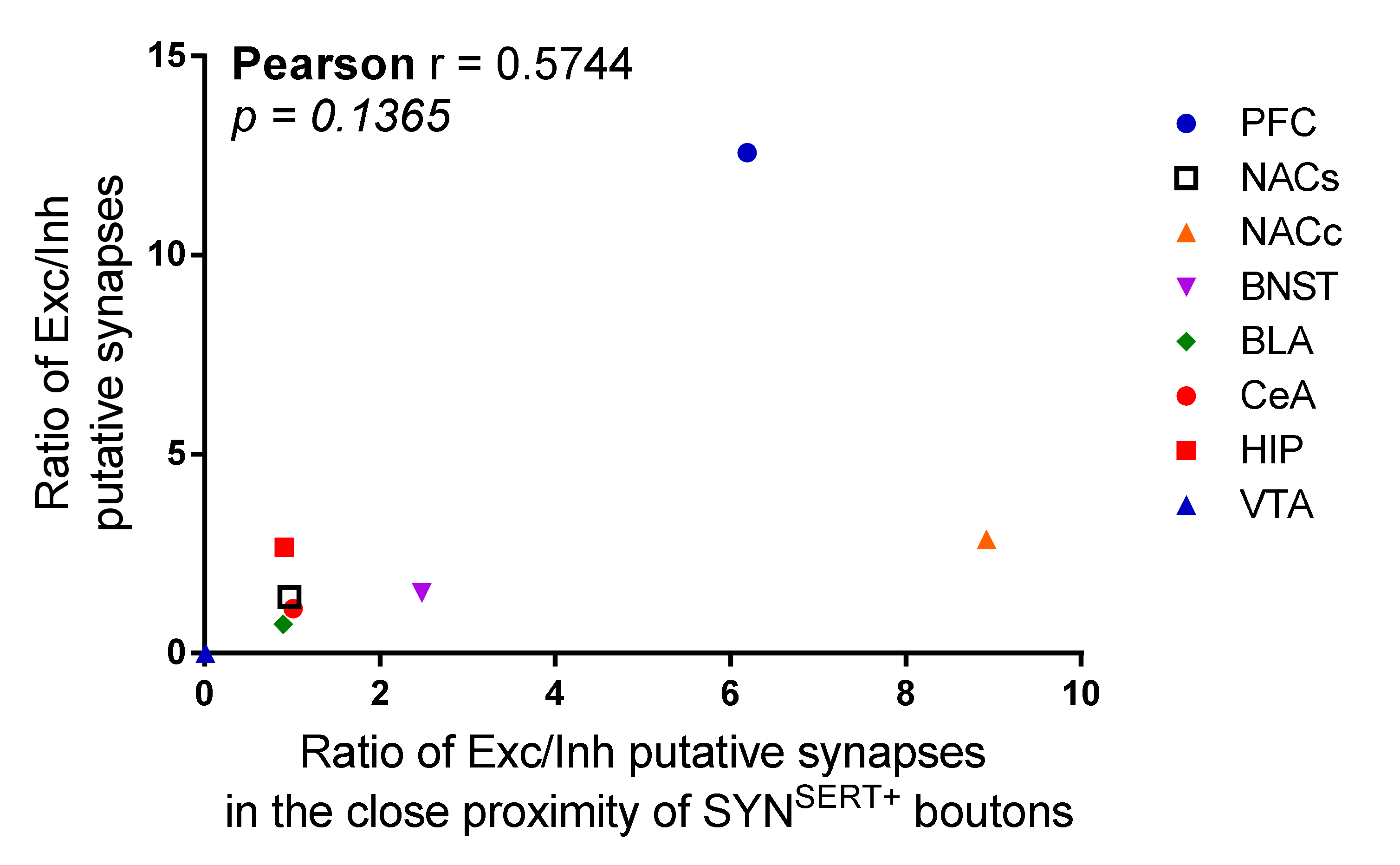

Supplement: Supplementary file 4 — Supplementary material 4 (TIFF 153 kb) [file 429_2016_1278_MOESM4_ESM.tif]
