# Supplementary material for: Mapping the connectivity of serotonin transporter immunoreactive axons to excitatory and inhibitory neurochemical synapses in the mouse limbic brain
Source: Brain Struct Funct. 2016 Aug 2;222(3):1297–314. doi: 10.1007/s00429-016-1278-x (PMC5368196; doi:10.1007/s00429-016-1278-x)
Supplement: Supplementary file 5 — Supplementary material 5 (DOCX 15 kb) [file 429_2016_1278_MOESM5_ESM.docx]

**Supplementary Figure legends**

**Fig. S1** Negative controls for first-stage immunohistochemistry. **a-d** Magnification showing the immunolabeling of the serotonin transporter SERT (green, **a)**, the presynaptic marker synaptophysin (blue, **b**), the postsynaptic marker of excitatory synapses PSD95 (purple, **c**) and the postsynaptic marker of inhibitory synapses gephyrin (red, **d**). **a’-d’** Micrographs of the non-specific labeling of respective negative controls, performed by omission of each primary antibody. *Scale bars*, **a-d** and **a’-d’**: 10 μm

**Fig. S2** Negative controls for second-stage immunohistochemistry. **a-c** Micrographs showing the immunolabeling of the serotonin transporter SERT (green) as the first step **(a)**. The non-specific labeling in the second step, performed by omission of the second primary antibody (gephyrin) following the first step immunolabeling of the serotonin transporter SERT and blockade of the free IgGs with normal rabbit serum and monovalent anti-rabbit F(ab) fragments (**b).** The second step immunolabeling of the postsynaptic marker of inhibitory synapses gephyrin (red), following the first step immunolabeling of the serotonin transporter SERT and blockade of the free IgGs with normal rabbit serum and monovalent anti-rabbit F(ab) fragments **(c)**. **d-f** Micrographs showing the immunolabeling of the presynaptic marker synaptophysin (blue) as the first step **(d)**. The non-specific labeling in the second step was performed by omission of the second primary antibody (synaptophysin) following the first step immunolabeling of PSD95 and blockade of the free IgGs with normal mouse serum and monovalent anti-mouse F(ab) fragments (**e).** Second step immunolabeling of the postsynaptic marker of excitatory synapses PSD95 (purple), after the first step immunolabeling of the presynaptic marker synaptophysin and blockade of the free IgGs with normal mouse serum and monovalent anti-mouse F(ab) fragments **(f)**. *Scale bars*, **a-c** and **d-f**: 10 μm

**Fig. S3** Removal of light-scattering by image deconvolution. Light scattering from confocal microscope acquisition might induce over-estimation of the fluorescence signal. **a** and **b** micrograph showing the comparison of raw vs deconvolved images of SERT^+^ fibers in the PFC (*Top scale bar:* 10 μm) . The bottom boxes are showing the SERT^+^ fibers in the z-plane (*Box scale bar:* 4μm). **c** and **d** micrograph the comparison of raw vs deconvolved images of PSD95 puncta in the PFC (*Top scale bar:* 10 μm). The bottom boxes are showing the PSD95 puncta in the z-plane (*Box scale bar:* 2μm). Comparison of **a**/**b** or **c**/**d** shows how deconvolution compensates for image distortion induced by light-scattering to allow for high resolution reconstruction of fibers and puncta images

**Fig. S4** Pearson correlation between the density of SYN/SERT^+^ boutons in the proximity of putative excitatory/inhibitory synapses and the density of putative excitatory/inhibitory synapses detected throughout the different brain regions. The absence of correlation (Pearson *r* = 0.5744, *p* = 0.1365) shows that the detection of SYN/SERT^+^ boutons in the proximity of putative excitatory/inhibitory synapses in each brain is not dependent on the density of putative excitatory/inhibitory synapses in each regions.
