# Supplementary material for: Mapping the connectivity of serotonin transporter immunoreactive axons to excitatory and inhibitory neurochemical synapses in the mouse limbic brain
Source: Brain Struct Funct. 2016 Aug 2;222(3):1297–314. doi: 10.1007/s00429-016-1278-x (PMC5368196; doi:10.1007/s00429-016-1278-x)
Supplement: Supplementary file 6 — Supplementary material 6 (DOCX 73 kb) [file 429_2016_1278_MOESM6_ESM.docx]

**Supp Table 1: Statistical analysis of SERT^+^ fiber density.**

| **Bonferroni's multiple comparisons test** | | **Mean Diff.** | **95% CI of diff.** | **P Value** | **Summary** |
| --- | --- | --- | --- | --- | --- |
| **PFC** | PFC vs. NAcS | -8.518 | -14.40 to -2.638 | **0.0002** | ******* |
|  | PFC vs. NAcC | -0.5087 | -6.388 to 5.371 | > 0.9999 | ns |
|  | PFC vs. BNST | -5.995 | -11.87 to -0.1153 | **0.0407** | ***** |
|  | PFC vs. CeA | -1.307 | -7.187 to 4.572 | > 0.9999 | ns |
|  | PFC vs. BLA | -13.51 | -19.39 to -7.626 | **< 0.0001** | ******** |
|  | PFC vs. HIP | -4.424 | -10.30 to 1.455 | 0.51 | ns |
|  | PFC vs. VTA | -48.72 | -54.60 to -42.84 | **< 0.0001** | ******** |
| **NACs** | NAcS vs. NAcC | 8.009 | 2.130 to 13.89 | **0.0007** | ******* |
|  | NAcS vs. BNST | 2.523 | -3.357 to 8.403 | > 0.9999 | ns |
|  | NAcS vs. CeA | 7.211 | 1.331 to 13.09 | **0.0039** | ****** |
|  | NAcS vs. BLA | -4.988 | -10.87 to 0.8917 | 0.2203 | ns |
|  | NAcS vs. HIP | 4.094 | -1.786 to 9.973 | 0.8056 | ns |
|  | NAcS vs. VTA | -40.2 | -46.08 to -34.32 | **< 0.0001** | ******** |
| **NACc** | NAcC vs. BNST | -5.486 | -11.37 to 0.3934 | 0.0984 | ns |
|  | NAcC vs. CeA | -0.7987 | -6.678 to 5.081 | > 0.9999 | ns |
|  | NAcC vs. BLA | -13 | -18.88 to -7.118 | **< 0.0001** | ******** |
|  | NAcC vs. HIP | -3.916 | -9.795 to 1.964 | > 0.9999 | ns |
|  | NAcC vs. VTA | -48.21 | -54.09 to -42.33 | **< 0.0001** | ******** |
| **BNST** | BNST vs. CeA | 4.688 | -1.192 to 10.57 | 0.3478 | ns |
|  | BNST vs. BLA | -7.511 | -13.39 to -1.631 | **0.0021** | ****** |
|  | BNST vs. HIP | 1.571 | -4.309 to 7.450 | > 0.9999 | ns |
|  | BNST vs. VTA | -42.73 | -48.61 to -36.85 | **< 0.0001** | ******** |
| **CeA** | CeA vs. BLA | -12.2 | -18.08 to -6.319 | **< 0.0001** | ******** |
|  | CeA vs. HIP | -3.117 | -8.997 to 2.763 | > 0.9999 | ns |
|  | CeA vs. VTA | -47.41 | -53.29 to -41.53 | **< 0.0001** | ******** |
| **BLA** | BLA vs. HIP | 9.082 | 3.202 to 14.96 | **< 0.0001** | ******** |
|  | BLA vs. VTA | -35.22 | -41.09 to -29.34 | **< 0.0001** | ******** |
| **HIP** | HIP vs. VTA | -44.3 | -50.18 to -38.42 | **< 0.0001** | ******** |

*: p<0.05 ; **: p<0.01; ***: p<0.001 and ****: p<0.0001, as compared to the brain regions mentioned, by One-way ANOVA analysis of variance followed by Bonferroni post-hoc comparisons. An overall effect was observed between the brain regions analysed, F(7, 232) = 150.7, p<0.0001.

**Supp Table 2: Statistical analysis of SERT^+^ fiber diameter (axon + bouton)**

| **Bonferroni's multiple comparisons test** | | **Mean Diff.** | **95% CI of diff.** | **P Value** | **Summary** |
| --- | --- | --- | --- | --- | --- |
| **PFC** | PFC vs. NAcS | -0.04367 | -0.0878 to 0.0005 | 0.057 | ns |
|  | PFC vs. NAcC | -0.04167 | -0.0858 to 0.0025 | 0.09 | ns |
|  | PFC vs. BNST | -0.00633 | -0.0505 to 0.0378 | > 0.9999 | ns |
|  | PFC vs. CeA | -0.067 | -0.1112 to -0.0227 | **< 0.0001** | ******** |
|  | PFC vs. BLA | -0.09633 | -0.1406 to -0.0521 | **< 0.0001** | ******** |
|  | PFC vs. HIP | -0.07 | -0.1142 to -0.0257 | **< 0.0001** | ******** |
|  | PFC vs. VTA | -0.148 | -0.1922 to -0.1038 | **< 0.0001** | ******** |
| **NACs** | NAcS vs. NAcC | 0.002 | -0.0422 to 0.0462 | > 0.9999 | ns |
|  | NAcS vs. BNST | 0.03733 | -0.0068 to 0.0815 | 0.2288 | ns |
|  | NAcS vs. CeA | -0.02333 | -0.0675 to 0.0208 | > 0.9999 | ns |
|  | NAcS vs. BLA | -0.05267 | -0.0968 to -0.0084 | **0.0059** | ****** |
|  | NAcS vs. HIP | -0.02633 | -0.0705 to 0.0178 | > 0.9999 | ns |
|  | NAcS vs. VTA | -0.1043 | -0.1486 to -0.0601 | **< 0.0001** | ******** |
| **NACc** | NAcC vs. BNST | 0.03533 | -0.0088 to 0.0795 | 0.3428 | ns |
|  | NAcC vs. CeA | -0.02533 | -0.0695 to 0.0188 | > 0.9999 | ns |
|  | NAcC vs. BLA | -0.05467 | -0.0988 to -0.0104 | **0.0034** | ****** |
|  | NAcC vs. HIP | -0.02833 | -0.0725 to 0.0158 | > 0.9999 | ns |
|  | NAcC vs. VTA | -0.1063 | -0.1506 to -0.0621 | **< 0.0001** | ******** |
| **BNST** | BNST vs. CeA | -0.06067 | -0.1049 to -0.0164 | **0.0006** | ******* |
|  | BNST vs. BLA | -0.09 | -0.1342 to -0.0457 | **< 0.0001** | ******** |
|  | BNST vs. HIP | -0.06367 | -0.1079 to -0.0194 | **0.0002** | ******* |
|  | BNST vs. VTA | -0.1417 | -0.1859 to -0.0974 | **< 0.0001** | ******** |
| **CeA** | CeA vs. BLA | -0.02933 | -0.0735 to 0.0148 | > 0.9999 | ns |
|  | CeA vs. HIP | -0.003 | -0.0472 to 0.0412 | > 0.9999 | ns |
|  | CeA vs. VTA | -0.081 | -0.1252 to -0.0367 | **< 0.0001** | ******** |
| **BLA** | BLA vs. HIP | 0.02633 | -0.0178 to 0.0705 | > 0.9999 | ns |
|  | BLA vs. VTA | -0.05167 | -0.0958 to -0.0074 | **0.0078** | ****** |
| **HIP** | HIP vs. VTA | -0.078 | -0.1222 to -0.0337 | **< 0.0001** | ******** |

*: p<0.05 ; **: p<0.01; ***: p<0.001 and ****: p<0.0001, as compared to the brain regions mentioned, by One-way ANOVA analysis of variance followed by Bonferroni post-hoc comparisons. An overall effect was observed between the brain regions analysed, F(7, 232) = 23.77, p<0.0001.

**Supp Table 3: Statistical analysis of the density of SYN^SERT+^ boutons per 10^3^ mm^3^ of tissue**

| **Bonferroni's multiple comparisons test** | | **Mean Diff.** | **95% CI of diff.** | **P Value** | **Summary** |
| --- | --- | --- | --- | --- | --- |
| **PFC** | PFC vs. NAcS | -8.736 | -21.43 to 3.954 | 0.8568 | ns |
|  | PFC vs. NAcC | 6.495 | -6.195 to 19.19 | > 0.9999 | ns |
|  | PFC vs. BNST | -3.067 | -15.76 to 9.623 | > 0.9999 | ns |
|  | PFC vs. CeA | 0.4567 | -12.23 to 13.15 | > 0.9999 | ns |
|  | PFC vs. BLA | -10.64 | -23.33 to 2.049 | 0.2409 | ns |
|  | PFC vs. HIP | 1.373 | -11.32 to 14.06 | > 0.9999 | ns |
|  | PFC vs. VTA | -90.13 | -102.8 to -77.44 | **< 0.0001** | ******** |
| **NACs** | NAcS vs. NAcC | 15.23 | 2.541 to 27.92 | **0.0053** | ****** |
|  | NAcS vs. BNST | 5.668 | -7.022 to 18.36 | > 0.9999 | ns |
|  | NAcS vs. CeA | 9.192 | -3.498 to 21.88 | 0.643 | ns |
|  | NAcS vs. BLA | -1.905 | -14.59 to 10.78 | > 0.9999 | ns |
|  | NAcS vs. HIP | 10.11 | -2.581 to 22.80 | 0.3498 | ns |
|  | NAcS vs. VTA | -81.39 | -94.08 to -68.70 | **< 0.0001** | ******** |
| **NACc** | NAcC vs. BNST | -9.563 | -22.25 to 3.127 | 0.5054 | ns |
|  | NAcC vs. CeA | -6.039 | -18.73 to 6.651 | > 0.9999 | ns |
|  | NAcC vs. BLA | -17.14 | -29.83 to -4.446 | **0.0008** | ******* |
|  | NAcC vs. HIP | -5.122 | -17.81 to 7.568 | > 0.9999 | ns |
|  | NAcC vs. VTA | -96.62 | -109.3 to -83.93 | **< 0.0001** | ******** |
| **BNST** | BNST vs. CeA | 3.524 | -9.166 to 16.21 | > 0.9999 | ns |
|  | BNST vs. BLA | -7.573 | -20.26 to 5.117 | > 0.9999 | ns |
|  | BNST vs. HIP | 4.441 | -8.249 to 17.13 | > 0.9999 | ns |
|  | BNST vs. VTA | -87.06 | -99.75 to -74.37 | **< 0.0001** | ******** |
| **CeA** | CeA vs. BLA | -11.1 | -23.79 to 1.593 | 0.1729 | ns |
|  | CeA vs. HIP | 0.9167 | -11.77 to 13.61 | > 0.9999 | ns |
|  | CeA vs. VTA | -90.59 | -103.3 to -77.90 | **< 0.0001** | ******** |
| **BLA** | BLA vs. HIP | 12.01 | -0.6760 to 24.70 | 0.086 | ns |
|  | BLA vs. VTA | -79.49 | -92.18 to -66.80 | **< 0.0001** | ******** |
| **HIP** | HIP vs. VTA | -91.5 | -104.2 to -78.81 | **< 0.0001** | ******** |

*: p<0.05 ; **: p<0.01; ***: p<0.001 and ****: p<0.0001, as compared to the brain regions mentioned, by One-way ANOVA analysis of variance followed by Bonferroni post-hoc comparisons. An overall effect was observed between the brain regions analysed, F(7, 232) = 124.2, p<0.0001.

**Supp Table 4: Statistical analysis of the density of SYN^SERT+^ boutons per 10^3^ mm^3^ of fiber.**

| **Bonferroni's multiple comparisons test** | | **Mean Diff.** | **95% CI of diff.** | **P Value** | **Summary** |
| --- | --- | --- | --- | --- | --- |
| **PFC** | PFC vs. NAcS | 0.2673 | -0.1093 to 0.6440 | 0.7231 | ns |
|  | PFC vs. NAcC | 1.19 | 0.8137 to 1.567 | **< 0.0001** | ******** |
|  | PFC vs. BNST | 0.5473 | 0.1707 to 0.9240 | **0.0002** | ******* |
|  | PFC vs. CeA | 0.3393 | -0.03729 to 0.7160 | 0.1345 | ns |
|  | PFC vs. BLA | 0.4643 | 0.08771 to 0.8410 | **0.0036** | ****** |
|  | PFC vs. HIP | 0.787 | 0.4104 to 1.164 | **< 0.0001** | ******** |
|  | PFC vs. VTA | -0.38 | -0.7566 to -0.0033 | **0.0455** | ***** |
| **NACs** | NAcS vs. NAcC | 0.923 | 0.5464 to 1.300 | **< 0.0001** | ******** |
|  | NAcS vs. BNST | 0.28 | -0.09662 to 0.6566 | 0.5498 | ns |
|  | NAcS vs. CeA | 0.072 | -0.3046 to 0.4486 | > 0.9999 | ns |
|  | NAcS vs. BLA | 0.197 | -0.1796 to 0.5736 | > 0.9999 | ns |
|  | NAcS vs. HIP | 0.5197 | 0.1430 to 0.8963 | **0.0005** | ******* |
|  | NAcS vs. VTA | -0.6473 | -1.024 to -0.2707 | **< 0.0001** | ******** |
| **NACc** | NAcC vs. BNST | -0.643 | -1.020 to -0.2664 | **< 0.0001** | ******** |
|  | NAcC vs. CeA | -0.851 | -1.228 to -0.4744 | **< 0.0001** | ******** |
|  | NAcC vs. BLA | -0.726 | -1.103 to -0.3494 | **< 0.0001** | ******** |
|  | NAcC vs. HIP | -0.4033 | -0.7800 to -0.02671 | 0.0234 | * |
|  | NAcC vs. VTA | -1.57 | -1.947 to -1.194 | **< 0.0001** | ******** |
| **BNST** | BNST vs. CeA | -0.208 | -0.5846 to 0.1686 | > 0.9999 | ns |
|  | BNST vs. BLA | -0.083 | -0.4596 to 0.2936 | > 0.9999 | ns |
|  | BNST vs. HIP | 0.2397 | -0.1370 to 0.6163 | > 0.9999 | ns |
|  | BNST vs. VTA | -0.9273 | -1.304 to -0.5507 | **< 0.0001** | ******** |
| **CeA** | CeA vs. BLA | 0.125 | -0.2516 to 0.5016 | > 0.9999 | ns |
|  | CeA vs. HIP | 0.4477 | 0.07104 to 0.8243 | **0.0061** | ****** |
|  | CeA vs. VTA | -0.7193 | -1.096 to -0.3427 | **< 0.0001** | ******** |
| **BLA** | BLA vs. HIP | 0.3227 | -0.05396 to 0.6993 | 0.2039 | ns |
|  | BLA vs. VTA | -0.8443 | -1.221 to -0.4677 | **< 0.0001** | ******** |
| **HIP** | HIP vs. VTA | -1.167 | -1.544 to -0.7904 | **< 0.0001** | ******** |

*: p<0.05 ; **: p<0.01; ***: p<0.001 and ****: p<0.0001, as compared to the brain regions mentioned, by One-way ANOVA analysis of variance followed by Bonferroni post-hoc comparisons. An overall effect was observed between the brain regions analysed, F(7, 232) = 31.99, p<0.0001.

**Supp Table 5: Statistical analysis of the density of total excitatory triads**

| **Bonferroni's multiple comparisons test** | | **Mean Diff.** | **95% CI of diff.** | **P Value** | **Summary** |
| --- | --- | --- | --- | --- | --- |
| **PFC** | PFC vs. NAcS | 180.4 | 94.01 to 266.8 | **< 0.0001** | ******** |
|  | PFC vs. NAcC | 289.4 | 203.0 to 375.8 | **< 0.0001** | ******** |
|  | PFC vs. BNST | 247.3 | 160.9 to 333.7 | **< 0.0001** | ******** |
|  | PFC vs. CeA | 76.37 | -10.01 to 162.8 | 0.1579 | ns |
|  | PFC vs. BLA | 105.4 | 18.98 to 191.8 | 0.0042 | ** |
|  | PFC vs. HIP | 195.4 | 109.0 to 281.8 | **< 0.0001** | ******** |
|  | PFC vs. VTA | 339.4 | 253.0 to 425.8 | **< 0.0001** | ******** |
| **NacS** | NAcS vs. NAcC | 109 | 22.63 to 195.4 | 0.0025 | ** |
|  | NAcS vs. BNST | 66.9 | -19.49 to 153.3 | 0.4237 | ns |
|  | NAcS vs. CeA | -104 | -190.4 to -17.63 | 0.0051 | ** |
|  | NAcS vs. BLA | -75.02 | -161.4 to 11.36 | 0.1829 | ns |
|  | NAcS vs. HIP | 14.97 | -71.41 to 101.4 | > 0.9999 | ns |
|  | NAcS vs. VTA | 159 | 72.65 to 245.4 | **< 0.0001** | ******** |
| **NacC** | NAcC vs. BNST | -42.12 | -128.5 to 44.26 | > 0.9999 | ns |
|  | NAcC vs. CeA | -213 | -299.4 to -126.7 | **< 0.0001** | ******** |
|  | NAcC vs. BLA | -184 | -270.4 to -97.66 | **< 0.0001** | ******** |
|  | NAcC vs. HIP | -94.05 | -180.4 to -7.661 | 0.0193 | * |
|  | NAcC vs. VTA | 50.01 | -36.37 to 136.4 | > 0.9999 | ns |
| **BNST** | BNST vs. CeA | -170.9 | -257.3 to -84.53 | **< 0.0001** | ******** |
|  | BNST vs. BLA | -141.9 | -228.3 to -55.53 | **< 0.0001** | ******** |
|  | BNST vs. HIP | -51.92 | -138.3 to 34.46 | > 0.9999 | ns |
|  | BNST vs. VTA | 92.14 | 5.750 to 178.5 | 0.0246 | * |
| **CeA** | CeA vs. BLA | 29 | -57.39 to 115.4 | > 0.9999 | ns |
|  | CeA vs. HIP | 119 | 32.61 to 205.4 | **0.0006** | ******* |
|  | CeA vs. VTA | 263.1 | 176.7 to 349.4 | **< 0.0001** | ******** |
| **BLA** | BLA vs. HIP | 90 | 3.611 to 176.4 | 0.0322 | * |
|  | BLA vs. VTA | 234.1 | 147.7 to 320.4 | **< 0.0001** | ******** |
| **HIP** | HIP vs. VTA | 144.1 | 57.67 to 230.4 | **< 0.0001** | ******** |

*: p<0.05 ; **: p<0.01; ***: p<0.001 and ****: p<0.0001, as compared to the brain regions mentioned, by one-way ANOVA analysis of variance followed by Bonferroni post-hoc comparisons. An overall effect was observed between the brain regions analysed, F(7, 232) = 34.74, p<0.0001.

**Supp Table 6: Statistical analysis of the density of pre- vs postsynaptic orientated excitatory triads**

| **Bonferroni's multiple comparisons test** | | **Mean Diff.** | **95% CI of diff.** | **P Value** | **Summary** |
| --- | --- | --- | --- | --- | --- |
| **PFC** | pre vs. post | 17.67 | -1.286 to 36.63 | 0.0768 | ns |
|  | pre vs. both | -142.2 | -161.1 to -123.2 | < 0.0001 | **** |
|  | post vs. both | -159.8 | -178.8 to -140.9 | < 0.0001 | **** |
| **NACs** | pre vs. post | 10.47 | -8.486 to 29.43 | 0.5553 | ns |
|  | pre vs. both | -90.59 | -109.5 to -71.63 | < 0.0001 | **** |
|  | post vs. both | -101.1 | -120.0 to -82.11 | < 0.0001 | **** |
| **NACc** | pre vs. post | 1.926 | -17.03 to 20.88 | > 0.9999 | ns |
|  | pre vs. both | -43.49 | -62.44 to -24.53 | < 0.0001 | **** |
|  | post vs. both | -45.41 | -64.37 to -26.46 | < 0.0001 | **** |
| **BNST** | pre vs. post | 2.756 | -16.20 to 21.71 | > 0.9999 | ns |
|  | pre vs. both | -51.91 | -70.86 to -32.95 | < 0.0001 | **** |
|  | post vs. both | -54.66 | -73.62 to -35.70 | < 0.0001 | **** |
| **CeA** | **pre vs. post** | **28.7** | **9.739 to 47.65** | **0.0009** | *** |
|  | pre vs. both | -123.4 | -142.4 to -104.4 | < 0.0001 | **** |
|  | post vs. both | -152.1 | -171.0 to -133.1 | < 0.0001 | **** |
| **BLA** | pre vs. post | 13.97 | -4.990 to 32.92 | 0.2321 | ns |
|  | pre vs. both | -84.76 | -103.7 to -65.81 | < 0.0001 | **** |
|  | post vs. both | -98.73 | -117.7 to -79.77 | < 0.0001 | **** |
| **HIP** | pre vs. post | 13.97 | -4.990 to 32.92 | 0.2321 | ns |
|  | pre vs. both | -84.76 | -103.7 to -65.81 | < 0.0001 | **** |
|  | post vs. both | -98.73 | -117.7 to -79.77 | < 0.0001 | **** |
| **VTA** | pre vs. post | 0.4767 | -18.48 to 19.43 | > 0.9999 | ns |
|  | pre vs. both | -0.111 | -19.07 to 18.85 | > 0.9999 | ns |
|  | post vs. both | -0.5877 | -19.54 to 18.37 | > 0.9999 | ns |

*: p<0.05 ; **: p<0.01; ***: p<0.001 and ****: p<0.0001, as compared to the pre-, postsynaptic orientated or equidistant, by two-way ANOVA analysis of variance followed by Bonferroni post-hoc comparisons. An overall interaction was observed between the 2 factors “brain region” x “triad orientation”: F(14, 464) = 29.52, p<0.0001, driven by main effects in both factors: “brain region”: F(7, 232) = 34.59, p< 0.001; “triad orientation”: F(2, 464) = 603.9, p<0.0001.

**Supp Table 7: Statistical analysis of the density of total inhibitory triads**

| **Bonferroni's multiple comparisons test** | | **Mean Diff.** | **95% CI of diff.** | **P Value** | **Summary** |
| --- | --- | --- | --- | --- | --- |
| **PFC** | PFC vs. NAcS | -144.6 | -275.5 to -13.66 | **0.0162** | ***** |
|  | PFC vs. NAcC | 37.29 | -93.61 to 168.2 | > 0.9999 | ns |
|  | PFC vs. BNST | -65.6 | -196.5 to 65.30 | > 0.9999 | ns |
|  | PFC vs. CeA | -352.3 | -483.2 to -221.4 | **< 0.0001** | ******** |
|  | PFC vs. BLA | -329 | -459.9 to -198.1 | **< 0.0001** | ******** |
|  | PFC vs. HIP | -40.87 | -171.8 to 90.03 | > 0.9999 | ns |
|  | PFC vs. VTA | -798.1 | -929.0 to -667.2 | **< 0.0001** | ******** |
| **NACs** | NAcS vs. NAcC | 181.9 | 50.95 to 312.8 | **0.0005** | ******* |
|  | NAcS vs. BNST | 78.96 | -51.94 to 209.9 | > 0.9999 | ns |
|  | NAcS vs. CeA | -207.7 | -338.6 to -76.80 | **< 0.0001** | ******** |
|  | NAcS vs. BLA | -184.4 | -315.3 to -53.54 | **0.0004** | ******* |
|  | NAcS vs. HIP | 103.7 | -27.22 to 234.6 | 0.3638 | ns |
|  | NAcS vs. VTA | -653.5 | -784.5 to -522.6 | **< 0.0001** | ******** |
| **NACc** | NAcC vs. BNST | -102.9 | -233.8 to 28.01 | 0.3835 | ns |
|  | NAcC vs. CeA | -389.6 | -520.5 to -258.7 | **< 0.0001** | ******** |
|  | NAcC vs. BLA | -366.3 | -497.2 to -235.4 | **< 0.0001** | ******** |
|  | NAcC vs. HIP | -78.17 | -209.1 to 52.74 | > 0.9999 | ns |
|  | NAcC vs. VTA | -835.4 | -966.3 to -704.5 | **< 0.0001** | ******** |
| **BNST** | BNST vs. CeA | -286.7 | -417.6 to -155.8 | **< 0.0001** | ******** |
|  | BNST vs. BLA | -263.4 | -394.3 to -132.5 | **< 0.0001** | ******** |
|  | BNST vs. HIP | 24.73 | -106.2 to 155.6 | > 0.9999 | ns |
|  | BNST vs. VTA | -732.5 | -863.4 to -601.6 | **< 0.0001** | ******** |
| **CeA** | CeA vs. BLA | 23.26 | -107.6 to 154.2 | > 0.9999 | ns |
|  | CeA vs. HIP | 311.4 | 180.5 to 442.3 | **< 0.0001** | ******** |
|  | CeA vs. VTA | -445.8 | -576.8 to -314.9 | **< 0.0001** | ******** |
| **BLA** | BLA vs. HIP | 288.1 | 157.2 to 419.0 | **< 0.0001** | ******** |
|  | BLA vs. VTA | -469.1 | -600.0 to -338.2 | **< 0.0001** | ******** |
| **HIP** | HIP vs. VTA | -757.2 | -888.1 to -626.3 | **< 0.0001** | ******** |

*: p<0.05 ; **: p<0.01; ***: p<0.001 and ****: p<0.0001, as compared to the brain regions mentioned, by one-way ANOVA analysis of variance followed by Bonferroni post-hoc comparisons. An overall effect was observed between the brain regions analysed, F(7, 232) = 89.80, p<0.0001.

**Supp Table 8: Statistical analysis of the density of pre- vs postsynaptic orientated inhibitory triads**

| **Bonferroni's multiple comparisons test** | | **Mean Diff.** | **95% CI of diff.** | **P Value** | **Summary** |
| --- | --- | --- | --- | --- | --- |
| **PFC** | pre vs. post | 9.88 | -20.43 to 40.19 | > 0.9999 | ns |
|  | pre vs. both | -2.841 | -33.15 to 27.47 | > 0.9999 | ns |
|  | post vs. both | -12.72 | -43.03 to 17.59 | 0.9412 | ns |
| **NACs** | **pre vs. post** | **32.34** | **2.027 to 62.64** | **0.032** | ***** |
|  | pre vs. both | -37.86 | -68.17 to -7.554 | 0.0085 | ** |
|  | post vs. both | -70.2 | -100.5 to -39.89 | < 0.0001 | **** |
| **NACc** | pre vs. post | 1.818 | -28.49 to 32.13 | > 0.9999 | ns |
|  | pre vs. both | -0.7867 | -31.09 to 29.52 | > 0.9999 | ns |
|  | post vs. both | -2.605 | -32.91 to 27.70 | > 0.9999 | ns |
| **BNST** | pre vs. post | 18.69 | -11.62 to 48.99 | 0.4176 | ns |
|  | pre vs. both | -17.08 | -47.39 to 13.23 | 0.5291 | ns |
|  | post vs. both | -35.77 | -66.07 to -5.458 | 0.0143 | * |
| **CeA** | pre vs. post | 26.52 | -3.787 to 56.83 | 0.1081 | ns |
|  | pre vs. both | -137 | -167.3 to -106.7 | < 0.0001 | **** |
|  | post vs. both | -163.5 | -193.8 to -133.2 | < 0.0001 | **** |
| **BLA** | pre vs. post | 23.44 | -6.869 to 53.75 | 0.1914 | ns |
|  | pre vs. both | -127.1 | -157.4 to -96.76 | < 0.0001 | **** |
|  | post vs. both | -150.5 | -180.8 to -120.2 | < 0.0001 | **** |
| **HIP** | pre vs. post | 23.44 | -6.869 to 53.75 | 0.1914 | ns |
|  | pre vs. both | -127.1 | -157.4 to -96.76 | < 0.0001 | **** |
|  | post vs. both | -150.5 | -180.8 to -120.2 | < 0.0001 | **** |
| **VTA** | pre vs. post | 7.987 | -22.32 to 38.29 | > 0.9999 | ns |
|  | pre vs. both | -368.5 | -398.8 to -338.2 | < 0.0001 | **** |
|  | post vs. both | -376.5 | -406.8 to -346.2 | < 0.0001 | **** |

*: p<0.05 ; **: p<0.01; ***: p<0.001 and ****: p<0.0001, as compared to the pre-, postsynaptic orientated or equidistant, by two-way ANOVA analysis of variance followed by Bonferroni post-hoc comparisons. An overall interaction was observed between the 2 factors “brain region” x “triad orientation”: F(14, 464) = 62.87, p<0.0001, driven by main effects in both factors: “brain region”: F (7, 232) = 75.32, p< 0.001; “triad orientation”: F (2, 464) = 423.2, p<0.0001.

**Supp Table 9: Statistical analysis of the density of extra-triadic boutons**

| **Bonferroni's multiple comparisons test** | | **Mean Diff.** | **95% CI of diff.** | **P Value** | **Summary** |
| --- | --- | --- | --- | --- | --- |
| **PFC** | PFC vs. NAcS | 1023 | 762.3 to 1283 | **< 0.0001** | ******** |
|  | PFC vs. NAcC | 886.4 | 626.0 to 1147 | **< 0.0001** | ******** |
|  | PFC vs. BNST | 409.7 | 149.2 to 670.1 | **< 0.0001** | ******** |
|  | PFC vs. CeA | 665.3 | 404.9 to 925.7 | **< 0.0001** | ******** |
|  | PFC vs. BLA | 705.2 | 444.8 to 965.6 | **< 0.0001** | ******** |
|  | PFC vs. HIP | 680.6 | 420.2 to 941.0 | **< 0.0001** | ******** |
|  | PFC vs. VTA | 160.2 | -100.2 to 420.6 | > 0.9999 | ns |
| **NACs** | NAcS vs. NAcC | -136.3 | -396.8 to 124.1 | > 0.9999 | ns |
|  | NAcS vs. BNST | -613.1 | -873.5 to -352.7 | **< 0.0001** | ******** |
|  | NAcS vs. CeA | -357.4 | -617.8 to -97.02 | **0.0006** | ******* |
|  | NAcS vs. BLA | -317.6 | -578.0 to -57.16 | **0.0042** | ****** |
|  | NAcS vs. HIP | -342.2 | -602.6 to -81.75 | **0.0013** | ****** |
|  | NAcS vs. VTA | -862.5 | -1123 to -602.1 | **< 0.0001** | ******** |
| **NACc** | NAcC vs. BNST | -476.7 | -737.1 to -216.3 | **< 0.0001** | ******** |
|  | NAcC vs. CeA | -221.1 | -481.5 to 39.33 | 0.219 | ns |
|  | NAcC vs. BLA | -181.2 | -441.6 to 79.19 | 0.8075 | ns |
|  | NAcC vs. HIP | -205.8 | -466.2 to 54.59 | 0.3693 | ns |
|  | NAcC vs. VTA | -726.2 | -986.6 to -465.8 | **< 0.0001** | ******** |
| **BNST** | BNST vs. CeA | 255.6 | -4.769 to 516.1 | 0.0604 | ns |
|  | BNST vs. BLA | 295.5 | 35.09 to 555.9 | **0.0115** | ***** |
|  | BNST vs. HIP | 270.9 | 10.49 to 531.3 | **0.0327** | ***** |
|  | BNST vs. VTA | -249.5 | -509.9 to 10.96 | 0.0769 | ns |
| **CeA** | CeA vs. BLA | 39.86 | -220.6 to 300.3 | > 0.9999 | ns |
|  | CeA vs. HIP | 15.26 | -245.2 to 275.7 | > 0.9999 | ns |
|  | CeA vs. VTA | -505.1 | -765.5 to -244.7 | **< 0.0001** | ******** |
| **BLA** | BLA vs. HIP | -24.59 | -285.0 to 235.8 | > 0.9999 | ns |
|  | BLA vs. VTA | -545 | -805.4 to -284.5 | **< 0.0001** | ******** |
| **HIP** | HIP vs. VTA | -520.4 | -780.8 to -260.0 | **< 0.0001** | ******** |

*: p<0.05 ; **: p<0.01; ***: p<0.001 and ****: p<0.0001, as compared to the brain regions mentioned, by One-way ANOVA analysis of variance followed by Bonferroni post-hoc comparisons. An overall effect was observed between the brain regions analysed, F (7, 232) = 36.32, p<0.0001.

**Supp Table 10: Statistical analysis of the excitatory/inhibitory ratio of triadic boutons**

| **Bonferroni's multiple comparisons test** | | **Mean Diff.** | **95% CI of diff.** | **P Value** | **Summary** |
| --- | --- | --- | --- | --- | --- |
| **PFC** | **exc vs. inh** | **296.9** | **174.0 to 419.9** | **< 0.0001** | ******** |
|  | exc vs extra-triadic | -726.2 | -849.2 to -603.3 | < 0.0001 | **** |
|  | inh vs extra-triadic | -1023 | -1146 to -900.2 | < 0.0001 | **** |
| **NACs** | exc vs. inh | -28.02 | -151.0 to 94.94 | > 0.9999 | ns |
|  | exc vs extra-triadic | 116.1 | -6.832 to 239.1 | 0.0711 | ns |
|  | inh vs extra-triadic | 144.1 | 21.19 to 267.1 | 0.0152 | * |
| **NACc** | exc vs. inh | 44.81 | -78.14 to 167.8 | > 0.9999 | ns |
|  | exc vs extra-triadic | -129.2 | -252.2 to -6.283 | 0.0357 | * |
|  | inh vs extra-triadic | -174.1 | -297.0 to -51.10 | 0.0022 | ** |
| **BNST** | exc vs. inh | -15.96 | -138.9 to 107.0 | > 0.9999 | ns |
|  | exc vs extra-triadic | -563.8 | -686.8 to -440.9 | < 0.0001 | **** |
|  | inh vs extra-triadic | -547.9 | -670.8 to -424.9 | < 0.0001 | **** |
| **CeA** | **exc vs. inh** | **-131.7** | **-254.7 to -8.746** | **0.0311** | ***** |
|  | exc vs extra-triadic | -137.3 | -260.2 to -14.33 | 0.0227 | * |
|  | inh vs extra-triadic | -5.585 | -128.5 to 117.4 | > 0.9999 | ns |
| **BLA** | **exc vs. inh** | **-137.4** | **-260.4 to -14.48** | **0.0225** | ***** |
|  | exc vs extra-triadic | -126.4 | -249.4 to -3.471 | 0.0416 | * |
|  | inh vs extra-triadic | 11.01 | -111.9 to 134.0 | > 0.9999 | ns |
| **HIP** | exc vs. inh | 60.69 | -62.26 to 183.7 | 0.7087 | ns |
|  | exc vs extra-triadic | -241 | -364.0 to -118.1 | < 0.0001 | **** |
|  | inh vs extra-triadic | -301.7 | -424.7 to -178.8 | < 0.0001 | **** |
| **VTA** | **exc vs. inh** | **-840.6** | **-963.6 to -717.6** | **< 0.0001** | **** |
|  | exc vs extra-triadic | -905.4 | -1028 to -782.5 | < 0.0001 | **** |
|  | inh vs extra-triadic | -64.84 | -187.8 to 58.12 | 0.6173 | ns |

*: p<0.05 ; **: p<0.01; ***: p<0.001 and ****: p<0.0001, as compared to the excitatory, inhibitory or extra-triadic SYN^SERT+^ boutons, by two-way ANOVA analysis of variance followed by Bonferroni post-hoc comparisons. An overall interaction was observed between the 2 factors “brain region” x “triad type”: F(14, 464) = 48.14, p<0.0001, driven by main effects in both factors: “brain region”: F(7, 232) = 43.46, p< 0.001; “triad type”: F(2, 464) = 187.4, p<0.0001.
